# Supplementary material for: Cohort study of neonatal resuscitation skill retention in frontline healthcare facilities in Bihar, India, after PRONTO simulation training
Source: BMJ Paediatr Open. 2020 Apr 20;4(1):e000628. doi: 10.1136/bmjpo-2019-000628 (PMC7204812; doi:10.1136/bmjpo-2019-000628)

BMJ Paediatrics Open

BMJ Paediatrics Open is committed to open peer review. As part of this commitment we make the peer review history of every article we publish publicly available.

When an article is published we post the peer reviewers' comments and the authors' responses online. We also post the versions of the paper that were used during peer review. These are the versions that the peer review comments apply to.

The versions of the paper that follow are the versions that were submitted during the peer review process. They are not the versions of record or the final published versions. They should not be cited or distributed as the published version of this manuscript.

BMJ Paediatrics Open is an open access journal and the full, final, typeset and author-corrected version of record of the manuscript is available on our site with no access controls, subscription charges or pay-per-view fees (http://bmjpaedsopen.bmj.com).

If you have any questions on BMJ Paediatrics Open's open peer review process please email info.bmjpo@bmj.com

BMJ Paediatrics Open

A cohort study of neonatal resuscitation skill retention in frontline health care facilities in Bihar, India after PRONTO simulation training

Journal:	*BMJ Paediatrics Open*
Manuscript ID	bmjpo-2019-000628
Article Type:	Original research
Date Submitted by the Author:	19-Dec-2019
Complete List of Authors:	Higgins, Brennan; University of California San Francisco, Pediatrics<br>Medvedev, Melissa; University of California San Francisco, Pediatrics<br>Spindler, Hilary; University of California San Francisco, Institute for Global Health Sciences<br>Ghosh, Rakesh; University of California San Francisco, Institute for Global Health Sciences<br>Longkumer, Ojungsangla; PRONTO International<br>Cohen, Susanna; University of Utah, College of Nursing<br>Das, Aritra; Care India<br>Gore, Aboli; Care India<br>Mahapatra, Tanmay; Care India<br>Walker, Dilys; University of California San Francisco, School of Medicine and Department of Obstetrics-Gynecology and Reproductive Sciences
Keywords:	Neonatology, Resuscitation

SCHOLARONE™
Manuscripts

I, the Submitting Author has the right to grant and does grant on behalf of all authors of the Work (as defined in the below author licence), an exclusive licence and/or a non-exclusive licence for contributions from authors who are: i) UK Crown employees; ii) where BMJ has agreed a CC-BY licence shall apply, and/or iii) in accordance with the terms applicable for US Federal Government officers or employees acting as part of their official duties; on a worldwide, perpetual, irrevocable, royalty-free basis to BMJ Publishing Group Ltd ("BMJ") its licensees and where the relevant Journal is co-owned by BMJ to the co-owners of the Journal, to publish the Work in this journal and any other BMJ products and to exploit all rights, as set out in our licence.

The Submitting Author accepts and understands that any supply made under these terms is made by BMJ to the Submitting Author unless you are acting as an employee on behalf of your employer or a postgraduate student of an affiliated institution which is paying any applicable article publishing charge ("APC") for Open Access articles. Where the Submitting Author wishes to make the Work available on an Open Access basis (and intends to pay the relevant APC), the terms of reuse of such Open Access shall be governed by a Creative Commons licence – details of these licences and which Creative Commons licence will apply to this Work are set out in our licence referred to above.

Other than as permitted in any relevant BMJ Author's Self Archiving Policies, I confirm this Work has not been accepted for publication elsewhere, is not being considered for publication elsewhere and does not duplicate material already published. I confirm all authors consent to publication of this Work and authorise the granting of this licence.

Confidential: For Review Only

A cohort study of neonatal resuscitation skill retention in frontline health care facilities in Bihar, India after PRONTO simulation training

Brennan V Higgins[1], Melissa M Medvedev[1,2], Hilary Spindler[3], Rakesh Ghosh[3], Ojungsangla Longkumer[4], Susanna R Cohen[5], Aritra Das[6], Aboli Gore[6], Tanmay Mahapatra[6], Dilys M Walker[7]

1. Department of Pediatrics, University of California San Francisco, 550 16th Street, 4th Floor, Box 0110, San Francisco, CA, 94158, USA
2. Maternal, Adolescent, Reproductive, and Child Health Centre, London School of Hygiene and Tropical Medicine, Keppel Street, London, WC1E 7HT, UK
3. Institute for Global Health Sciences, University of California San Francisco, 550 16th Street, San Francisco, CA 94158, USA
4. PRONTO International, State RMNCH+A, Patna, 80002, India
5. College of Nursing, University of Utah, 10 South 2000 East, Salt Lake City, UT, 84112, USA
6. CARE India, 14 Patliputra Colony, Patna, 800013, Bihar, India
7. School of Medicine and Department of Obstetrics-Gynecology and Reproductive Sciences, University of California San Francisco, 1001 Potrero Ave, San Francisco, CA, 94110, USA

Corresponding author: Brennan V Higgins
Address: Department of Pediatrics, University of California San Francisco, 550 16th Street, 4th Floor, Box 0110, San Francisco, CA 94143, USA
E-mail: brennan.vail@ucsf.edu
Telephone: 415-476-5001

Confidential: For Review Only

ABSTRACT

Background

Neonatal resuscitation (NR) simulation training programs have increased throughout low- and middle-income countries. Many of such programs have demonstrated a positive impact on NR knowledge and skill acquisition in addition to early neonatal mortality and fresh stillbirth rates. However, NR skill retention after simulation programs remains an ongoing challenge.

Methods

This study assessed facility level NR skill retention after PRONTO International's simulation training in Bihar, India. Training was conducted within CARE India's AMANAT mentoring program as part of a larger maternal and child health quality improvement initiative. Primary health centers (PHCs) were initially offered PRONTO training with AMANAT mentoring, facilitated by bachelors trained nurses, during eight-month phases between September 2015 and January 2017. Repeat training began in February 2018 and was facilitated by peers. NR skills in simulated resuscitations were assessed at the facility level at the mid- and endpoint of initial training and prior to and at the midpoint of repeat training.

Results

The percentage of facilities in which effective positive pressure ventilation (PPV) was delivered (31.1%) and infant heart rate was assessed (13.1%) increased from mid to post initial training (p=0.03). This was followed by a 26.2% and 20.9% decline in these skills respectively over the training gap ($p \leq 0.01$). A significant increase in heart rate assessment was again observed by the midpoint of repeat training (16.1%, $p = 0.04$). No significant change was observed in other skills assessed.

Conclusions

Despite an initial facility level improvement in select NR skills, skill deterioration was observed at a facility level post training. Given the technical nature of NR skills and the departure that these skills represent from traditional practices in Bihar, frequent refresher trainings at shorter intervals are likely necessary. Employing peer simulation facilitators may address the problem of sustainability that arises with increased training frequency.

What is known about the subject

- Neonatal resuscitation simulation training programs have spread throughout low- and middle-income settings; most with a model of brief periods (1-3 days) of instruction
- These training programs can improve provider knowledge and skill and thereby reduce early neonatal mortality and fresh stillbirths
- Skill retention after such training programs has been achieved only in programs with ongoing opportunities for training and practice

What this study adds

- This study evaluates skill retention after simulation training carried out over an eight-month period with the hypothesis that a longer duration of training may improve retention
- Despite a longer duration of initial training skill deterioration was still observed post-training

Confidential: For Review Only

- Nevertheless peer simulation facilitation proved to be an effective way to help frontline providers begin to re-build skills during refresher trainings

INTRODUCTION

Neonatal mortality represents a disproportionate and unsolved public health problem globally [1], and intrapartum-related events remain a leading cause [2]. In response, neonatal resuscitation (NR) simulation training programs have proliferated in low- and middle-income countries (LMICs) [3, 4, 5]. Nevertheless, skills of frontline providers remain a well-recognized barrier to immediate neonatal care [6] and a focus of the Every Newborn Action Plan, which aims to reduce neonatal mortality to 10 per 1000 live births in every country globally by 2035 [7]. Although NR training programs have already had a documented positive impact on knowledge and skill acquisition with subsequent reduction in early neonatal mortality and fresh stillbirths, skill retention after these programs remains a unique challenge and a key obstacle to the 2035 goal [3, 4, 5].

In LMICs, studies evaluating skill retention after NR simulation training programs have assessed retention from one to 12 months post-training. The majority of these studies assessed Helping Babies Breathe (HBB), a one to three-day simulation-based training program developed specifically for LMIC settings [8]. Other simulation training programs assessed were of comparable duration and content to HBB. Most of these studies reported some decline in NR skills after training completion, including at one month [9], three months [10], four months [11], and six months [12, 13, 14] post-training.

Notably, however, three studies did not observe uniform skill decay. One study observed maintenance of skills at nine to 12 months post-training, which the authors attributed to the unique study setting—a small group of providers (n=14) in a high-risk referral hospital in Accra, Ghana where providers perform NR daily [15]. A second study found a falloff in skills in two of three training groups at three to six months but maintenance of skills in the third group at this same timepoint [14]. In this latter group, simulators were left at the study facilities for ongoing practice, which was facilitated by clinical mentors who received phone support from district trainers. A final study saw improvements at three and 12-months post-training in all skills except positive pressure ventilation (PPV) [16]. In this study, facilities received the initial HBB training (two days) in addition to bi-weekly visits from trainers to encourage practice over the post-training period (12 months). Common to all three studies was the opportunity for frequent and ongoing practice. However, PPV was highlighted as an exception, with an observed decline in competence despite ongoing practice [16].

PRONTO International [17] developed a unique in-situ, intrapartum simulation training curriculum for LMIC settings, which was implemented in Bihar, India over a long initial training period of approximately eight months. The training was embedded in a statewide mentoring program called "Apatkaleen Matritva evam Navjat Tatparta" (AMANAT) and implemented by CARE India in partnership with Government of Bihar. Assessment of PRONTO training implemented with AMANAT mentoring has demonstrated improved NR skills post-training in both simulated and live deliveries [18], despite the presence of many structural, logistical, and cultural barriers to neonatal care [19, 20]. However, NR skill retention following PRONTO training, or any simulation training of comparable duration in a LMIC setting, has not been

Confidential: For Review Only

assessed. The aim of this study was to assess retention of NR skills after PRONTO simulation training within the AMANAT mentoring program in Bihar for programmatic evaluation and possible application in similar LMIC settings.

METHODS

Study setting and population

Training was conducted in Bihar, a state in eastern India, which is the third most populous in the country [21]. According to the World Bank, more than one-third of Bihar's population lives below the international poverty line (<$1.90 per day) [22]. The documented neonatal mortality rate is 37 per 1000 live births [23], with significant under-reporting likely. This study assesses PRONTO training conducted alongside AMANAT mentoring in government basic emergency obstetric and neonatal care (BEmONC) facilities which were largely primary health centers (PHCs). PHCs serve as entry-level facilities for preventative and basic medical care. In PHCs, the majority of deliveries are attended by Auxiliary Nurse Midwives (ANM) or General Nurse Midwives (GNM), frontline providers with two or three and a half years of training after general education, respectively [24].

AMANAT and PRONTO interventions

AMANAT was a statewide on the job mentoring program for the obstetric and newborn care providers in public sector facilities. The program was implemented by CARE India [25] in collaboration with the government of Bihar as part of a health-system strengthening and quality of care improvement effort. PRONTO International [17] provided simulation training on obstetric and neonatal emergencies within the broader AMANAT initiative using the AMANAT mentor/mentee structure for clinical instruction. PRONTO training consists of in-situ, intrapartum simulation training in addition to teamwork and communication exercises, skill stations, and case-based learning. The intrapartum nature of PRONTO training emphasizes care of the mother-infant dyad, including complications that may co-exist such as NR and postpartum hemorrhage [26]. However, skills assessed in this study were based on performance in NR simulations without co-existing maternal complications.

This study assessed PRONTO training conducted within the AMANAT initiative for BEmONC facilities (PHCs) between September 2015 and February 2019. From September 2015 to January 2017, training/mentoring was conducted in three approximately eight-month phases (AMANAT phases two through four, Figure 1). AMANAT phase one was excluded due to curricular and simulation assessment differences during that initial phase. In phases two through four, training was conducted at 80 non-overlapping PHCs per phase, or a total of 240 PHCs. Phase five, which began in February 2018, marked the initiation of repeat PRONTO training at PHCs through a second chapter of the AMANAT initiative called AMANAT Jyoti, which is ongoing. The focus of AMANAT Jyoti is on sustainability and continued health system strengthening. Phase five reached 353 PHCs statewide. The majority of these PHCs had received prior training/mentoring in phases one through four, but a few were not included in the original simulation training and mentoring program. Notably, even if the PHC had received prior simulation training and mentoring, due to staff turnover not all ANMs/GNMs in a given PHC during phase five had participated in prior training. The exact percentage of providers who had received prior training is unknown.

Confidential: For Review Only

In AMANAT phases two through four, PRONTO NR simulations were facilitated by AMANAT nurse mentors who were college educated (BSc and MSc) nurses recruited from across India. Using a train-the-trainer approach, PRONTO staff provided a six-day initial training course on NR and simulation techniques for the nurse mentors followed by a four-day refresher training that occurred three months into each phase. Mentees who received the PRONTO simulation curriculum were ANMs and GNMs at PHCs. Nurse mentors worked in pairs under the supervision of a master nurse mentor to facilitate PRONTO's NR simulations at four PHCs, spending one week per month at each PHC. NR simulations were conducted beginning in month three of training and continued through the end of the phase (month eight). However, NR clinical instruction and mentorship during live deliveries began at the outset of training. A single NR assessment simulation was conducted in each PHC at month four (mid-assessment) and month eight (post-assessment) of training to assess acquisition of NR skills amongst mentees at a facility level. Pre-training assessments were not conducted to give mentees time to adjust to simulation-based learning and thereby minimize the potential impact of simulation artifact on data. In phases two and three, the simulated NR assessment scenario began immediately after birth, whereas in phase four, the scenario also included a normal spontaneous vaginal delivery. All NR assessment simulations were video-recorded for immediate debriefing and program assessment purposes.

In phase 5, with the focus of AMANAT Jyoti shifted towards sustainability, mentors responsible for simulation facilitation transitioned to a cadre of ANMs and GNMs working in the Bihar PHC system. These new mentors received the same initial six-day PRONTO training; however, training was delivered by previous AMANAT nurse mentors rather than PRONTO staff. New ANM/GNM mentors were then responsible for PRONTO simulation facilitation with peer ANM/GNM mentees in PHCs. Prior to phase five, from February to April 2018, a single NR assessment simulation was conducted in a random sample of PHCs (n=105) by PRONTO staff to assess skill retention at the facility level from prior training in phases two through four. Following baseline assessments, NR training was then introduced between July and October 2018. Subsequently, mid-assessments were conducted between October 2018 and February 2019 to assess NR skill acquisition under the new peer mentorship model. Similar to phase four, baseline and mid-assessment simulations in phase five began with a normal spontaneous vaginal delivery and subsequently required NR. Simulations were video-recorded for debriefing and skill assessment.

Ethics approval

Ethical approval for this study was granted from the Committee on Human Research at UCSF (14-15446) and the Institutional Committee for Ethics and Review of the Indian Institute of Health Management Research. All mentees provided consent for the use of video data in aggregated analyses.

Patient and public involvement

The data for this manuscript was simulation data and participants were frontline healthcare providers. Patients did not participate in this research. Results will be disseminated to local facilities via PRONTO International and CARE India to assist with quality of care improvement efforts.

Confidential: For Review Only

Analysis

NR assessment simulation videos from all phases were analyzed by a Neonatal Resuscitation Program [27] certified physician at the University of California San Francisco (UCSF). Due to the minor differences in the simulated clinical scenario during different phases (with or without a preceding delivery), data could not be coded in a single blinded group. Phase two through three mid- and post-assessment simulations were coded in one blinded group. Phase four mid- and post-assessments and phase five baseline assessments were coded in a second blinded group. Data from phase five mid-assessments was available at a later date and thus was coded separately.

Assessment data was matched by facility across time to assess NR skill acquisition and retention at the facility level. Timepoints were defined as follows (Figure 1):

A) Phase two, three, or four mid-assessment
B) Phase two, three, or four post-assessment
C) Phase five baseline assessment
D) Phase five mid-assessment

The training gap between timepoints B and C allowed for assessment of NR skill retention at a facility level. The duration of this gap ranged from 12 to 20 months depending on the initial training phase (Figure 1). A Cochran's Q test was conducted to assess if there was any difference at any timepoint in the percentage of facilities in which key NR skills were correctly performed at timepoints A through C across matched facilities. Due to the matched nature of this test, only facilities where skills could be evaluated at all timepoints were included in the statistical analysis. The number of facilities included varied by skill as not all skills could be evaluated in every simulation video due to video quality issues. For skills that demonstrated a statistically significant change over time, McNemar's test was used to individually assess change from timepoint A to B, timepoint B to C, and timepoint A to C. Additionally, a McNemar's test was conducted to assess skill acquisition at the facility level from timepoint C to D in phase five under the new peer mentorship model. All statistical analyses were conducted using SPSS 23 [28].

RESULTS

NR assessment simulations were conducted at all timepoints A through C at 64 PHCs across Bihar. This included 24 PHCs initially trained in phase two, 14 in phase three, and 26 in phase four. A significant increase in the percentage of facilities in which two key NR skills-- PPV with chest rise and heart rate assessment-- were performed correctly was observed over the initial training period (timepoint A to B) followed by a decline over the training gap (timepoint B to C) (Table 1, Figure 2, $p<0.01$). For PPV with chest rise, a 31.1 percentage-point increase from mid- to post-initial training/mentoring was observed (timepoint A to B, $p=0.03$) followed by a 26.2 percentage-point decrease from the initial training post-assessment to the repeat training baseline assessment (timepoint B to C, $p=0.01$). For assessment of heart rate, an initial 13.1 percentage-point increase from mid- to post-training (timepoint A to B, $p=0.03$) was followed by a 20.9 percentage-point decrease over the training gap (timepoint B to C, $p<0.01$).

Although not statistically significant, the percentage of facilities in which chest rise was achieved in ≤30 seconds increased from post-initial training (51.2%) to baseline reassessment (68.6%)

(timepoint B to C, Figure 2). In all other skills assessed including stimulation, suction, neck extension, and rate of PPV delivery, there was no statistically significant change over time (timepoints A through C). Finally, there was no statistically significant change from initial mid-assessment (timepoint A) to baseline re-assessment (timepoint C) for all skills including PPV with chest rise and heart rate assessment.

Of the 64 facilities that completed assessments at all timepoints A through C, 40 had a fourth assessment at timepoint D. Additionally, five facilities that received training/mentoring for the first time in phase five, participated in both phase five baseline assessments (timepoint C) and mid-training assessments (timepoint D). Amongst these 45 facilities with data at both timepoints C and D, a significant increase was observed in the percentage of facilities in which heart rate was assessed from baseline (79.5%) to mid repeat training (95.6%) (Table 2, Figure 3, $p = 0.04$). There were no significant changes in the remaining skills assessed including stimulation, suction, neck extension, PPV with chest rise, and rate of PPV delivery (Table 2, Figure 3).

DISCUSSION

To be effective, NR requires not only knowledge of the resuscitation algorithm, but also technical competence in performing PPV and awareness of the need for continual re-evaluation of a neonate's clinical status, including assessment of heart rate. With initial PRONTO training, as part of the larger AMANAT nurse mentoring interventions in Bihar, an increase was observed in the proportion of PHCs in which PPV was delivered with effective chest rise and heart rate was assessed, consistent with a previous assessment conducted in 2017 [18]. However, this initial facility-level improvement was followed by a decline in these same skills over a prolonged training gap. Nevertheless, under the new mentorship model of peer simulation facilitation employed in repeat training as part of AMANAT Jyoti, the proportion of PHCs in which heart rate was assessed significantly improved again.

To maintain technical NR skills, such as PPV, frequent repetition is key. Similar to this study, other studies have shown a decline in NR skills at one to six months post training without such repetition [9-14]. Programs that have achieved skill retention have in common the opportunity for frequent and ongoing NR practice through the time of skill re-assessment [14-16]. This study emphasizes this need for frequent NR skill practice amongst frontline providers, who may not have these opportunities in their daily clinical work, despite the eight-month duration of initial PRONTO training within the AMANAT mentoring program. This need for repetition in NR training was underscored by AMANAT mentors in previous qualitative interviews due to the fact that evidence-based NR practices represent a significant departure from traditional clinical practices in Bihar [19, 20]. PPV and heart rate assessment are perhaps the most unfamiliar and technical skills of basic NR, thus it is not surprising that these skills were the ones to degrade over the training gap [29]. In a previous study, AMANAT mentors also identified supply issues, including availability of appropriately sized masks for ventilation bags and watches for keeping time, as additional barriers to PPV and heart rate assessment [18]. Although all necessary supplies were provided during simulation, lack of supply availability during the training gap may have contributed to the observed facility level skill decline by limiting practice opportunities.

Among the other skills assessed, no significant changes were observed at the facility level across all four timepoints. It is possible that this was due to inadequate sample size, nevertheless, this

Confidential: For Review Only

pattern is overall consistent with a prior program assessments and hypotheses regarding this lack of change are discussed in detail elsewhere [18]. It is important to note that baseline assessments were not conducted prior to initial PRONTO training in AMANAT phases two through four. This was a deliberate choice to avoid bias from simulation artifact given mentees' lack of familiarity with simulation-based learning. However, based on previous qualitative analyses of mentors' evaluations of mentees' NR skills prior to training [18], it is likely that the initial mid-training assessments (timepoint A) represent a significant improvement compared to true baseline. Therefore, the lack of change observed in this study, particularly from mid initial training (timepoint A) to re-assessment after the training gap (timepoint C), presumably reflects maintenance of acquired skills rather than lack of skill acquisition. Nevertheless, there is undoubtedly room for continued improvement.

This study had several limitations. As noted above, the small sample size may have precluded identification of significant changes in the performance of all NR skills. Additionally, this analysis assessed skill at a facility level. Due to high staff turnover at PHCs, not all ANM and GNM mentees participating in phase five had previously received PRONTO training in phases two through four. As previously stated, the exact percentage of phase five mentees who had received prior training was unknown. Assessing skill retention at the facility-level with this limitation risks over-estimating skill decline if nurses not previously trained enter PHCs in which training has already occurred. Another limitation was the difference in the beginning of the simulated NR scenario in phases two and three, where the scenario started after birth, versus in phases four and five, where the scenario started with a normal delivery. To reduce the risk of bias, only skills required in both clinical scenarios were assessed and the time in which those skills were completed was not assessed despite the importance of urgency in NR. Finally, PRONTO simulation training was implemented as part of the AMANAT mentoring program as previously described. While we are unable to distinguish the individual impact of simulation and mentoring on performance in assessment simulations, this is not dissimilar to other simulation training programs which involve more components than simulation alone. This assessment is meant to be an assessment of the PRONTO program within the context of AMANAT mentoring.

The optimal frequency of basic NR simulation training for frontline providers in LMIC settings remains unknown [30, 31], with no current consensus except that it should occur more frequently than annually [30]. It is estimated that 5-10% of neonates will require basic resuscitation to transition after birth and, of that group, 3-6% will require bag-mask ventilation [32]. For frontline providers, this translates into very few opportunities to practice technical NR skills. Therefore, annual training is likely insufficient. Two recent studies on cardiopulmonary resuscitation (CPR), a similarly rare event requiring competency in technical skills, suggested repeat training at one-month intervals was optimal [33, 34]. In one study, providers were randomized to receive refresher training at one, three, six, or 12-month intervals, and significantly improved performance was observed in the group that received monthly training compared to all other groups. Moreover, there was no difference in skill retention amongst groups who received refresher training at three, six, or 12-month intervals [34]. A monthly training interval is additionally supported by the fact that one month is the earliest published evidence of NR skill decline post simulation training in the LMIC literature [9]. Further, initial PRONTO training within AMANAT mentoring in Bihar occurred at monthly intervals over an

Confidential: For Review Only

eight-month training period and, at this interval, was demonstrated to improve NR skills in simulated and live deliveries [18].

Monthly training in an LMIC setting presents the unique challenge of sustainability. One possible solution is peer simulation facilitation. In this study, peer simulation facilitation was introduced in phase five with AMANAT Jyoti and was demonstrated to enable re-acquisition of heart rate assessment skills by the midpoint of training. Similarly, in Uganda peer-facilitated NR practice after an initial HBB training was demonstrated to promote maintenance of NR skills at six months post-training when peer facilitators received phone support from HBB trainers [14]. Finally, a randomized control trial in Syria demonstrated non-inferiority of peer-facilitated Basic Life Support training, which includes CPR, as compared to professionally-facilitated training [35]. Although more research is required into how to optimally train frontline healthcare providers to become experts in both clinical NR skills and simulation facilitation, limited existing evidence suggests peer instruction may offer both a successful and sustainable model of clinical instruction.

CONCLUSION

PRONTO simulation training implemented in Bihar as part of CARE India's AMANAT mentoring program in partnership with the local government, had a significant, positive impact on the delivery of effective PPV and neonatal heart rate assessment at the facility level. However, after a prolonged training gap, improvements in these skills were lost despite the relatively long duration of initial simulation training. Similar NR skill decline has been observed as early as one-month post-training in other LMIC settings, concordant with the limited opportunities frontline providers have to practice the technical skills required in NR in such settings. More frequent training is necessary and is especially important in settings where evidence-based NR skills may represent a large shift in clinical practice. In such settings, a key challenge is the scale and sustainability of any NR training program, especially if monthly training is indicated. Employing peer simulation facilitators may be one way to address these issues.

List of abbreviations

NR: neonatal resuscitation
LMIC: low- and middle-income country
HBB: Helping Babies Breathe
PPV: positive pressure ventilation
BEmONC: basic emergency obstetric and neonatal care
PHC: primary health center
ANM: auxiliary nurse midwife
GNM: general nurse midwife
UCSF: University of California San Francisco
CPR: cardiopulmonary resuscitation

Funding

This study was supported by the Bill and Melinda Gates Foundation, grant number OPP1112431. The funding body had no role in study design, data collection, analysis, interpretation, manuscript writing, or the decision to submit the manuscript for publication.

Confidential: For Review Only

Competing Interests
DW and SC are founding members of PRONTO International and sit on its board of directors. None of the other author have any competing interests to declare.

Authors' Contributions
BH led study design for this manuscript, analyzed all simulation videos, performed the data analysis, and drafted and revised the manuscript. MM and HS contributed to study design, literature review, data interpretation and critical manuscript revision. RG, OL, and SC were also involved study design, data interpretation and provided critical manuscript revision. RG additionally provided input in data analysis and OL additionally provided on the ground oversight of training and local contextualization. AD, AG and TM were involved in study design, implementation, provided local contextualization, and provided critical input during manuscript revision. DW was the principal investigator and a major contributor to all aspects of this study and manuscript.

Availability of data and material
Data is not yet publicly available as the study is ongoing. Requests for data/materials may be made to the corresponding author.

Acknowledgements
The authors would like to thank CARE India as well as the nurse supervisors, nurse mentors and mentees working in the primary health centers throughout the state of Bihar for their hard work implementing and participating in both the AMANAT and AMANAT Jyoti nurse mentoring programs. Thank you also to the PRONTO Patna based staff members for facilitating simulation and team trainings and implementing the PRONTO curriculum in both programs as well as collecting, organizing, and managing hundreds of simulation videos. Lastly, we would like to thank Dr. Hemant Shah and the rest of the CARE India management for their unrelenting efforts to improve conditions for women and newborns in Bihar.

Key Words
Neonatal resuscitation
Simulation
Skill retention
Bihar
India

REFERENCES

1. Collaborators GM. Global, regional, and national under-5 mortality, adult mortality, age-specific mortality, and life expectancy, 1970-2016: a systematic analysis for the Global Burden of Disease Study 2016. Lancet. 2017;390(10100):1084-150.
2. Liu L, Oza S, Hogan D, Chu Y, Perin J, Zhu J, et al. Global, regional, and national causes of under-5 mortality in 2000-15: an updated systematic analysis with implications for the Sustainable Development Goals. Lancet. 2016;388(10063):3027-35.

Confidential: For Review Only

Confidential: For Review Only

3. Reisman J, Arlington L, Jensen L, Louis H, Suarez-Rebling D, Nelson BD. Newborn Resuscitation Training in Resource-Limited Settings: A Systematic Literature Review. Pediatrics. 2016;138(2).
4. Kamath-Rayne BD, Berkelhamer SK, Kc A, Ersdal HL, Niermeyer S. Neonatal resuscitation in global health settings: an examination of the past to prepare for the future. Pediatr Res. 2017;82(2):194-200.
5. Dol J, Campbell-Yeo M, Murphy GT, Aston M, McMillan D, Richardson B. The impact of the Helping Babies Survive program on neonatal outcomes and health provider skills: a systematic review. JBI Database System Rev Implement Rep. 2018;16(3):701-37.
6. Enweronu-Laryea C, Dickson KE, Moxon SG, Simen-Kapeu A, Nyange C, Niermeyer S, et al. Basic newborn care and neonatal resuscitation: a multi-country analysis of health system bottlenecks and potential solutions. BMC Pregnancy Childbirth. 2015;15 Suppl 2:
7. WHO, UNICEF. 2014. *Every Newborn: an action plan to end preventable deaths*. Geneva: World Health Organization
8. Helping Babies Breathe. Available from: www.helpingbabiesbreathe.org [Accessed 15 March 2019].
9. Mildenberger C, Ellis C, Lee K. Neonatal resuscitation training for midwives in Uganda: Strengthening skill and knowledge retention. Midwifery. 2017;50:36-41.
10. Musafili A, Essén B, Baribwira C, Rukundo A, Persson L. Evaluating Helping Babies Breathe: training for healthcare workers at hospitals in Rwanda. Acta Paediatr. 2013;102(1):e34-8.
11. Eblovi D, Kelly P, Afua G, Agyapong S, Dante S, Pellerite M. Retention and use of newborn resuscitation skills following a series of helping babies breathe trainings for midwives in rural Ghana. Glob Health Action. 2017;10(1):1387985.
12. Carlo WA, Wright LL, Chomba E, McClure EM, Carlo ME, Bann CM, et al. Educational impact of the neonatal resuscitation program in low-risk delivery centers in a developing country. J Pediatr. 2009;154(4):504-8.e5.
13. Bang A, Patel A, Bellad R, Gisore P, Goudar SS, Esamai F, et al. Helping Babies Breathe (HBB) training: What happens to knowledge and skills over time? BMC Pregnancy Childbirth. 2016;16(1):364.
14. Evans CL, Bazant E, Atukunda I, Williams E, Niermeyer S, Hiner C, et al. Peer-assisted learning after onsite, low-dose, high-frequency training and practice on simulators to prevent and treat postpartum hemorrhage and neonatal asphyxia: A pragmatic trial in 12 districts in Uganda. PLoS One. 2018;13(12):e0207909.
15. Bookman L, Engmann C, Srofenyoh E, Enweronu-Laryea C, Owen M, Randolph G, et al. Educational impact of a hospital-based neonatal resuscitation program in Ghana. Resuscitation. 2010;81(9):1180-2.
16. Arabi AM, Ibrahim SA, Ahmed SE, MacGinnea F, Hawkes G, Dempsey E, et al. Skills retention in Sudanese village midwives 1 year following Helping Babies Breathe training. Arch Dis Child. 2016;101(5):439-42.
17. PRONTO International. Available from: www.prontointernational.org [Accessed 15 March 2019].
18. Vail B, Morgan MC, Spindler H, Christmas A, Cohen SR, Walker DM. The power of practice: simulation training improving the quality of neonatal resuscitation skills in Bihar, India. BMC Pediatr. 2018;18(1):291.

19. Vail B, Morgan MC, Dyer J, Christmas A, Cohen SR, Joshi M, et al. Logistical, cultural, and structural barriers to immediate neonatal care and neonatal resuscitation in Bihar, India. BMC Pregnancy Childbirth. 2018;18(1):385.
20. Morgan MC, Dyer J, Abril A, Christmas A, Mahapatra T, Das A, et al. Barriers and facilitators to the provision of optimal obstetric and neonatal emergency care and to the implementation of simulation-enhanced mentorship in primary care facilities in Bihar, India: a qualitative study. BMC Pregnancy Childbirth. 2018;18(1):420.
21. Registrar General of India. Population Enumeration Data. Available from: www.censusindia.gov.in/2011census/population_enumeration.html [Accessed 15 March 2019].
22. World Bank Group. Bihar: Poverty, Growth & Inequality. 2016 Available from: http://documents.worldbank.org/curated/en/781181467989480762/pdf/105842-BRI-P157572-PUBLIC-Bihar-Proverty.pdf [Accessed 15 March 2019].
23. Government of India Ministry of Health and Family Welfare. National Family Health Survey – 4. Available from: http://rchiips.org/nfhs/ [Accessed 15 March 2019].
24. Indian Nursing Council. Types of Nursing Programs. Available from: www.indiannursingcouncil.org/nursing-programs.asp?show=prog-type [Accessed 15 March 2019].
25. CARE India. Available from: www.careindia.org [Accessed 15 March 2019].
26. Vail B, Spindler H, Morgan MC, Cohen SR, Christmas A, Sah P, et al. Care of the mother-infant dyad: a novel approach to conducting and evaluating neonatal resuscitation simulation training in Bihar, India. BMC Pregnancy Childbirth. 2017;17(1):252.
27. Neonatal Resuscitation Program. www.aap.org/en-us/continuing-medical-education/life-support/NRP/Pages/NRP.aspx [Accessed 15 March 2019].
28. IBM Corp. Released 2013. IBM SPSS statistics for Macintosh, version 23.0. Armonk: IBM Corp.
29. Sawyer T, White M, Zaveri P, Chang T, Ades A, French H, et al. Learn, see, practice, prove, do, maintain: an evidence-based pedagogical framework for procedural skill training in medicine. Acad Med. 2015;90(8):1025-33.
30. Perlman JM, Wyllie J, Kattwinkel J, Wyckoff MH, Aziz K, Guinsburg R, et al. Part 7: Neonatal Resuscitation: 2015 International Consensus on Cardiopulmonary Resuscitation and Emergency Cardiovascular Care Science With Treatment Recommendations (Reprint). Pediatrics. 2015;136 Suppl 2:S120-66.
31. Sawyer T, Ades A, Ernst K, Colby C. Simulation and the Neonatal Resuscitation Program 7th Edition Curriculum. NeoReviews. 2016;17:e447.
32. Lee AC, Cousens S, Wall SN, Niermeyer S, Darmstadt GL, Carlo WA, et al. Neonatal resuscitation and immediate newborn assessment and stimulation for the prevention of neonatal deaths: a systematic review, meta-analysis and Delphi estimation of mortality effect. BMC Public Health. 2011;11(Suppl 3):S12.
33. Lin Y, Cheng A, Grant VJ, Currie GR, Hecker KG. Improving CPR quality with distributed practice and real-time feedback in pediatric healthcare providers - A randomized controlled trial. Resuscitation. 2018;130:6-12.
34. Anderson R, Sebaldt A, Lin Y, Cheng A. Optimal training frequency for acquisition and retention of high-quality CPR skills: A randomized trial. Resuscitation. 2019;135:153-61.

Confidential: For Review Only

35. Abbas F, Sawaf B, Hanafi I, Hajeer MY, Zakaria MI, Abbas W, et al. Peers versus professional training of basic life support in Syria: a randomized controlled trial. BMC Med Educ. 2018;18(1):142.

Confidential: For Review Only

TABLES and FIGURES

Table 1. Percentage of facilities in which key skills were correctly performed in simulated neonatal resuscitations at mid-PRONTO training, post-training, and following a 12 to 20-month training gap (N = 192 assessment simulations at 64 facilities)

NR Skill	N[1]	Timepoint A[2]	Timepoint B[3]	Timepoint C[4]		Δ A -> B[7]	Δ B -> C[7]	Δ A -> C[7]
		% (n)[5]			*p*-value[6]	Δ %		
Stimulation	60	55.6 (35)	50.8 (31)	55.6 (35)	0.92	-4.8	4.8	0
Suction	58	63.9 (39)	67.8 (40)	67.2 (43)	0.82	3.9	-0.6	3.3
Neck extension	49	78.2 (43)	80.7 (46)	80.6 (50)	0.68	2.5	-0.1	2.4
PPV with chest rise	41	58.9 (33)	90.0 (45)	63.8 (37)	0.01	31.1*	-26.2*	4.9
Chest rise in ≤30 seconds	14	53.1 (17)	51.2 (22)	68.6 (24)	0.46	-1.9	17.4	15.5
Rate of PPV 40-60 bpm	53	36.7 (22)	41.4 (24)	38.7 (24)	0.88	4.7	-2.7	2.0
Heart rate assessed	54	85.2 (52)	98.3 (57)	77.4 (48)	<0.01	13.1*	-20.9*	-7.8

NR neonatal resuscitation; *PPV* positive pressure ventilation; *bpm* beats per minute

1. Total number of facilities in which NR skill could be assessed at all three timepoints A-C, n varies due to video quality
2. Timepoint A = AMANAT phase 2, 3 or 4 mid-training assessment
3. Timepoint B = AMANAT phase 2, 3 or 4 post-training assessment
4. Timepoint C = AMANAT Jyoti phase 5 baseline assessment
5. Percent (number) of facilities in which NR skill was performed correctly at a given timepoint
6. Cochran's Q test
7. * = significant change based on McNemar's test, p-values reported in text

Table 2. Percentage of facilities in which key skills were correctly performed in simulated neonatal resuscitations at baseline and mid-repeat PRONTO training with peer simulation facilitators (N = 90 assessment simulations at 45 facilities)

NR Skill	N[1]	Timepoint C[2]	Timepoint D[3]	Δ C -> D	
		% (n)[4]		Δ %	*p*-value[5]

Stimulation	44	52.3 (23)	62.2 (28)	9.9	0.45
Suction	45	71.1 (32)	66.7 (30)	-4.4	0.84
Neck extension	43	77.3 (34)	90.9 (40)	13.6	0.11
PPV with chest rise	43	65.1 (28)	77.8 (35)	12.7	0.15
Chest rise in ≤30s	21	65.4 (17)	46.9 (15)	-18.5	0.11
Rate of PPV 40-60 bpm	43	41.9 (18)	51.1 (23)	9.2	0.38
Heart rate assessed	44	79.5 (35)	95.6 (43)	16.1	0.04

NR neonatal resuscitation; *PPV* positive pressure ventilation; *bpm* beats per minute

1. Total number of facilities in which NR skill could be assessed at both timepoints C and D, n varies due to video quality
2. Timepoint C = AMANAT Jyoti phase 5 baseline assessment
3. Timepoint D = AMANAT Jyoti phase 5 mid-training assessment
4. Percent (number) of facilities in which NR skill was performed correctly at a given timepoint
5. McNemar's test

Figure 1. Timing of AMANAT and AMANAT Jyoti training phases

Figure 2. Facility level NR skill acquisition and retention with PRONTO training

Figure 3. Facility level NR skill acquisition with peer-facilitated PRONTO training

Confidential: For Review Only

Figure 1. Timing of AMANAT and AMANAT Jyoti training phases

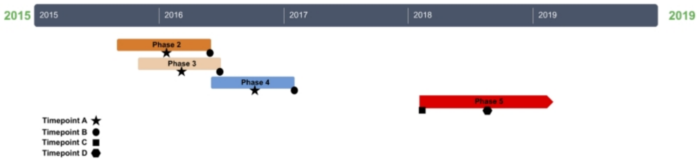

215x279mm (300 x 300 DPI)

Figure 2. Facility level NR skill acquisition and retention with PRONTO training

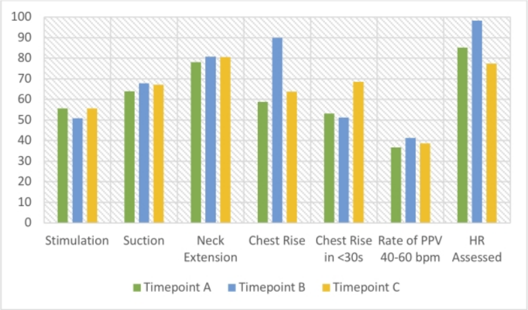

Timepoint A = AMANAT phase 2, 3 or 4 mid-training assessment
Timepoint B = AMANAT phase 2, 3 or 4 post-training assessment
Timepoint C = AMANAT Jyoti phase 5 baseline assessment

215x279mm (300 x 300 DPI)

Figure 3. Facility level NR skill acquisition with peer-facilitated PRONTO training

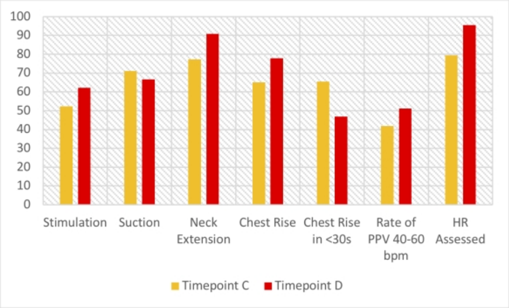

Timepoint C = AMANAT Jyoti phase 5 baseline assessment
Timepoint D = AMANAT Jyoti phase 5 mid-training assessment

215x279mm (300 x 300 DPI)

BMJ Paediatrics Open

A cohort study of neonatal resuscitation skill retention in frontline health care facilities in Bihar, India after PRONTO simulation training

Journal:	*BMJ Paediatrics Open*
Manuscript ID	bmjpo-2019-000628.R1
Article Type:	Original research
Date Submitted by the Author:	14-Feb-2020
Complete List of Authors:	Higgins, Brennan; University of California San Francisco, Pediatrics<br>Medvedev, Melissa; University of California San Francisco, Pediatrics<br>Spindler, Hilary; University of California San Francisco, Institute for Global Health Sciences<br>Ghosh, Rakesh; University of California San Francisco, Institute for Global Health Sciences<br>Longkumer, Ojungsangla; PRONTO International<br>Cohen, Susanna; University of Utah, College of Nursing<br>Das, Aritra; Care India<br>Gore, Aboli; Care India<br>Mahapatra, Tanmay; Care India<br>Walker, Dilys; University of California San Francisco, School of Medicine and Department of Obstetrics-Gynecology and Reproductive Sciences
Keywords:	Neonatology, Resuscitation

SCHOLARONE™
Manuscripts

I, the Submitting Author has the right to grant and does grant on behalf of all authors of the Work (as defined in the below author licence), an exclusive licence and/or a non-exclusive licence for contributions from authors who are: i) UK Crown employees; ii) where BMJ has agreed a CC-BY licence shall apply, and/or iii) in accordance with the terms applicable for US Federal Government officers or employees acting as part of their official duties; on a worldwide, perpetual, irrevocable, royalty-free basis to BMJ Publishing Group Ltd ("BMJ") its licensees and where the relevant Journal is co-owned by BMJ to the co-owners of the Journal, to publish the Work in this journal and any other BMJ products and to exploit all rights, as set out in our licence.

The Submitting Author accepts and understands that any supply made under these terms is made by BMJ to the Submitting Author unless you are acting as an employee on behalf of your employer or a postgraduate student of an affiliated institution which is paying any applicable article publishing charge ("APC") for Open Access articles. Where the Submitting Author wishes to make the Work available on an Open Access basis (and intends to pay the relevant APC), the terms of reuse of such Open Access shall be governed by a Creative Commons licence – details of these licences and which Creative Commons licence will apply to this Work are set out in our licence referred to above.

Other than as permitted in any relevant BMJ Author's Self Archiving Policies, I confirm this Work has not been accepted for publication elsewhere, is not being considered for publication elsewhere and does not duplicate material already published. I confirm all authors consent to publication of this Work and authorise the granting of this licence.

Confidential: For Review Only

A cohort study of neonatal resuscitation skill retention in frontline health care facilities in Bihar, India after PRONTO simulation training

Brennan V Higgins[1], Melissa M Medvedev[1,2], Hilary Spindler[3], Rakesh Ghosh[3], Ojungsangla Longkumer[4], Susanna R Cohen[5], Aritra Das[6], Aboli Gore[6], Tanmay Mahapatra[6], Dilys M Walker[7]

1. Department of Pediatrics, University of California San Francisco, 550 16th Street, 4th Floor, Box 0110, San Francisco, CA, 94158, USA
2. Maternal, Adolescent, Reproductive, and Child Health Centre, London School of Hygiene and Tropical Medicine, Keppel Street, London, WC1E 7HT, UK
3. Institute for Global Health Sciences, University of California San Francisco, 550 16th Street, San Francisco, CA 94158, USA
4. PRONTO International, State RMNCH+A, Patna, 80002, India
5. College of Nursing, University of Utah, 10 South 2000 East, Salt Lake City, UT, 84112, USA
6. CARE India, 14 Patliputra Colony, Patna, 800013, Bihar, India
7. School of Medicine and Department of Obstetrics-Gynecology and Reproductive Sciences, University of California San Francisco, 1001 Potrero Ave, San Francisco, CA, 94110, USA

Corresponding author: Brennan V Higgins
Address: Department of Pediatrics, University of California San Francisco, 550 16th Street, 4th Floor, Box 0110, San Francisco, CA 94143, USA
E-mail: brennan.vail@ucsf.edu
Telephone: 415-476-5001

Confidential: For Review Only

ABSTRACT

Background

Neonatal resuscitation (NR) simulation training programs have increased throughout low- and middle-income countries. Many of such programs have demonstrated a positive impact on NR knowledge and skill acquisition and early neonatal mortality and fresh stillbirth rates. However, NR skill retention after simulation programs remains a challenge.

Methods

This study assessed facility level NR skill retention after PRONTO International's simulation training in Bihar, India. Training was conducted within CARE India's AMANAT mentoring program as part of a larger maternal and child health quality improvement initiative. Primary health centres (PHCs) were initially offered PRONTO training with AMANAT mentoring, facilitated by bachelor trained nurses, during eight-month phases between September 2015 and January 2017. Repeat training began in February 2018 and was facilitated by peers. NR skills in simulated resuscitations were assessed at the facility level at the mid- and endpoint of initial training and prior to and at the midpoint of repeat training.

Results

The percentage of facilities in which effective positive pressure ventilation (PPV) was delivered and infant heart rate was assessed increased from mid- to post-initial training (31.1% and 13.1% respectively, p=0.03; n=64 PHCs and 192 simulations). This was followed by a 26.2% and 20.9% decline in these skills respectively over the training gap ($p \leq 0.01$). A significant increase in heart rate assessment was again observed by the midpoint of repeat training with peer facilitators (16.1%, p = 0.04; n=45 PHCs and 90 simulations). No change was observed in other skills assessed.

Conclusions

Despite an initial facility level improvement in select NR skills, deterioration was observed at a facility level post training. Given the technical nature of NR skills and the departure that these skills represent from traditional practices in Bihar, frequent refresher trainings at shorter intervals are likely necessary. Employing peer simulation facilitators may enable such increased training frequency.

What is known about the subject

- Neonatal resuscitation simulation training programs have spread throughout low- and middle-income settings; most with a model of brief periods (1-3 days) of instruction
- These training programs can improve provider knowledge and skill and thereby reduce early neonatal mortality and fresh stillbirths
- Skill retention after such training programs has been achieved only in programs with ongoing opportunities for training and practice

What this study adds

- This study evaluates skill retention after simulation training carried out over an eight-month period with the hypothesis that a longer duration of training may improve retention

Confidential: For Review Only

- Despite a longer duration of initial training, skill deterioration was still observed post-training
- Refresher trainings were possible with peer simulation facilitators and with further study could be an effective model for providing more frequent simulation training in frontline facilities

INTRODUCTION

Neonatal mortality represents a disproportionate and unsolved public health problem globally [1], and intrapartum-related events remain a leading cause [2]. In response, neonatal resuscitation (NR) simulation training programs have proliferated in low- and middle-income countries (LMICs) [3, 4, 5]. Nevertheless, skills of frontline providers remain a well-recognized barrier to immediate neonatal care [6] and a focus of the Every Newborn Action Plan, which aims to reduce neonatal mortality to 10 per 1000 live births in every country globally by 2035 [7]. Although NR training programs have already had a documented positive impact on knowledge and skill acquisition with subsequent reduction in early neonatal mortality and fresh stillbirths, skill retention after these programs remains a unique challenge and a key obstacle to the 2035 goal [3, 4, 5].

In LMICs, studies evaluating skill retention after NR simulation training programs have assessed retention from one to 12 months post-training. The majority of these studies assessed Helping Babies Breathe (HBB), a one to three-day simulation-based training program developed specifically for LMIC settings [8]. Other simulation training programs assessed were of comparable duration and content to HBB. Most of these studies reported some decline in NR skills after training completion, including at one month [9], three months [10], four months [11], and six months [12, 13, 14] post-training.

Notably, however, three studies did not observe uniform skill decay. One study observed maintenance of skills at nine to 12 months post-training, which the authors attributed to the unique study setting—a small group of providers (n=14) in a high-risk referral hospital in Accra, Ghana where providers perform NR daily [15]. A second study found a falloff in skills in two of three training groups at three to six months but maintenance of skills in the third group at this same timepoint [14]. In this latter group, simulators were left at the study facilities for ongoing practice, which was facilitated by clinical mentors who received phone support from district trainers. A final study saw improvements at three and 12-months post-training in all skills except positive pressure ventilation (PPV) [16]. In this study, facilities received the initial HBB training (two days) in addition to bi-weekly visits from trainers to encourage practice over the post-training period (12 months). Common to all three studies was the opportunity for frequent and ongoing practice. However, PPV was highlighted as an exception, with an observed decline in competence despite ongoing practice [16].

PRONTO International [17] developed a unique in-situ, intrapartum simulation training curriculum for LMIC settings, which was implemented in Bihar, India over a long initial training period of approximately eight months. The training was embedded in a state-wide mentoring program called "Apatkaleen Matritva evam Navjat Tatparta" (AMANAT) and implemented by CARE India in partnership with Government of Bihar. Assessment of PRONTO training implemented with AMANAT mentoring has demonstrated improved NR skills post-training in

Confidential: For Review Only

both simulated and live deliveries [18], despite the presence of many structural, logistical, and cultural barriers to neonatal care [19, 20]. However, NR skill retention following PRONTO training, or any simulation training of comparable duration in a LMIC setting, has not been assessed. The aim of this study was to assess retention of NR skills after PRONTO simulation training within the AMANAT mentoring program in Bihar for programmatic evaluation and possible application in similar LMIC settings. A secondary objective of this study was to assess skill re-acquisition with peer-facilitated PRONTO simulations to better understand the feasibility of this approach to simulation training.

Confidential: For Review Only

METHODS

Study setting and population

Training was conducted in Bihar, a state in Northeastern India, which is the third most populous in the country [21]. According to the World Bank, more than one-third of Bihar's population lives below the international poverty line (<$1.90 per day) [22]. The documented neonatal mortality rate is 37 per 1000 live births [23], with significant under-reporting likely. This study assessed PRONTO training conducted alongside AMANAT mentoring in government basic emergency obstetric and neonatal care (BEmONC) facilities which were largely primary health centres (PHCs).

PHCs serve as entry-level facilities for preventative and basic medical care. AMANAT reached 353 PHCs across Bihar. Approximately 1.3 million deliveries are conducted annually in these PHCs [24]. The delivery load per facility varies by location. Approximately 10% of mothers are referred from PHCs to higher-level facilities for delivery [24]. Deliveries in PHCs are attended by Auxiliary Nurse Midwives (ANM) or General Nurse Midwives (GNM), frontline providers with two or three and a half years of training after general education, respectively [25]. ANM/GNMs perform a variety of tasks at PHCs in addition to labour and delivery care. These include other clinical obligations, including emergency care and outpatient care, as well as clerical tasks such as taking supply inventory and completing maternity registers. ANM/GNMs also perform community outreach for immunisations roughly twice per week. The average out-of-pocket expenditure of mothers in 2019 for a PHC delivery was 1788 Indian rupees or 25 United States dollars. The majority of expenses were collected for diagnostic tests [26].

AMANAT and PRONTO interventions

AMANAT was a state-wide on the job mentoring program for the obstetric and newborn care providers in public sector facilities. The program was implemented by CARE India [27] in collaboration with the government of Bihar as part of a health-system strengthening and quality of care improvement effort. PRONTO International [17] provided simulation training on obstetric and neonatal emergencies within the broader AMANAT initiative using the AMANAT mentor/mentee structure for clinical instruction. PRONTO training consists of in-situ, intrapartum simulation training in addition to teamwork and communication exercises, skill stations, and case-based learning. The intrapartum nature of PRONTO training emphasises care of the mother-infant dyad, including complications that may co-exist such as NR and postpartum haemorrhage [28]. However, skills assessed in this study were based on performance in NR simulations without co-existing maternal complications.

This study assessed PRONTO training conducted within the AMANAT initiative at PHCs between September 2015 and February 2019. The timeline of the intervention is depicted in Figure 1 and described below. From September 2015 to January 2017, PRONTO training was conducted in three equivalent eight-month phases (AMANAT phases two through four). AMANAT phase one was excluded due to curricular and simulation assessment differences during that initial phase. Each phase reached 80 non-overlapping PHCs for a total of 240 PHCs. This was followed by a training gap prior to initiation of repeat PRONTO training as part of a second chapter of the AMANAT initiative called AMANAT Jyoti, which is ongoing.

The focus of AMANAT Jyoti is on sustainability and continued health system strengthening. This second round of PRONTO training within AMANAT Jyoti reached 353 PHCs state-wide. The majority of these PHCs had received prior training/mentoring during AMANAT and thus AMANAT Jyoti marked the initiation of repeat PRONTO training. However, a few PHCs were not included in the original PRONTO training during AMANAT mentoring. Notably, even if the PHC had received prior simulation training and mentoring, due to staff turnover not all ANMs/GNMs in a given PHC had participated in the prior training. The exact percentage of providers who had received prior training is unknown.

In AMANAT, PRONTO NR simulations were facilitated by AMANAT nurse mentors who were college educated (BSc and MSc) nurses recruited from across India. Using a train-the-trainer approach, PRONTO staff provided a six-day initial training course on NR and simulation techniques for the nurse mentors followed by a four-day refresher training that occurred three months into each phase. Mentees who received the PRONTO simulation curriculum were ANMs and GNMs at PHCs. ANM/GNMs were not paid to participate in PRONTO training or AMANAT mentoring and this was their first experience with simulation-based NR training. Some ANM/GNM mentees had participated in other governmental classroom-based trainings on topics including basic newborn care and NR.

Nurse mentors worked in pairs under the supervision of a master nurse mentor to facilitate PRONTO's NR simulations at four PHCs, spending one week per month at each PHC. As depicted in Figure 1, NR simulations were conducted beginning in month three of training and continued monthly through the end of the phase (month 8). However, NR clinical instruction and mentorship during live deliveries began at the outset of training. A single NR assessment simulation was conducted in each PHC at month four (AMANAT mid-assessment) and month eight (AMANAT post-assessment) of training to assess acquisition of NR skills amongst mentees at a facility level. Pre-training assessments were not conducted to give mentees time to adjust to simulation-based learning and thereby minimise the potential impact of simulation artefact on data. In AMANAT phases two and three, the simulated NR assessment scenario began immediately after birth, whereas in phase four, the scenario also included a normal spontaneous vaginal delivery. All NR assessment simulations were video-recorded for immediate debriefing and program assessment purposes.

With the focus of AMANAT Jyoti shifted towards sustainability, mentors responsible for simulation facilitation transitioned to a cadre of peer ANMs and GNMs working in the Bihar PHC system (Figure 1). These new mentors received the same initial six-day PRONTO training. New ANM/GNM mentors were then responsible for PRONTO simulation facilitation with peer

Confidential: For Review Only

ANM/GNM mentees in PHCs. As depicted in Figure 1, a single NR baseline re-assessment simulation (AMANAT Jyoti baseline re-assessment) was conducted in a random sample of PHCs (n=105) by PRONTO staff prior to initiation of repeat PRONTO training to assess skill retention at the facility level from AMANAT post-assessments. Following baseline assessments, NR simulation training was then introduced between July and October 2018. One simulation was conducted per PHC during this interval. Subsequently, AMANAT Jyoti mid re-assessments were conducted between October 2018 and February 2019 to assess NR skill acquisition with the new peer mentorship model. Both AMANAT Jyoti baseline re-assessment and mid re-assessment simulations began with a normal spontaneous vaginal delivery and subsequently required NR. Simulations were video-recorded for debriefing and skill assessment.

Ethics approval

Ethical approval for this study was granted from the Committee on Human Research at University of California San Francisco (UCSF; 14-15446) and the Institutional Committee for Ethics and Review of the Indian Institute of Health Management Research. All mentees provided consent for the use of video data in aggregated analyses.

Patient and public involvement

The data for this manuscript was simulation data and participants were frontline healthcare providers. Patients did not participate in this research. Results will be disseminated to local facilities via PRONTO International and CARE India to assist with quality of care improvement efforts.

Analysis

NR assessment simulation videos from all phases were analysed by a Neonatal Resuscitation Program [29] certified physician at UCSF. Due to the minor differences in the simulated clinical scenario (with or without a preceding delivery), data could not be coded in a single blinded group. Assessment data was matched by facility across time to assess NR skill acquisition and retention at the facility level. The four assessment timepoints per PHC were AMANAT mid assessments, AMANAT post assessments, AMANAT Jyoti baseline re-assessments, and AMANANAT Jyoti mid re-assessments. The training gap between AMANAT post assessments and AMANAT Jyoti baseline re-assessments allowed for assessment of NR skill retention at a facility level. The duration of this gap ranged from 15 to 29 months depending on the AMANAT phase in which the PHC originally participated (Figure 1).

A Cochran's Q test was conducted to assess if there was any difference across matched facilities in the percentage of facilities in which key NR skills were correctly performed at any time from AMANAT mid assessments to AMANAT Jyoti baseline re-assessments. Due to the matched nature of this test, only facilities where skills could be evaluated at all timepoints (mid assessment, post assessment, and baseline re-assessment) were included in the statistical analysis. The number of facilities included varied by skill as not all skills could be evaluated in every simulation video due to video quality issues. For skills that demonstrated a statistically significant change over time, McNemar's test was used to individually assess change from AMANAT mid to post assessments, AMANAT post assessments to AMANAT Jyoti baseline re-assessments, and AMANAT mid assessments to AMANAT Jyoti baseline re-assessments.

Confidential: For Review Only

Additionally, a McNemar's test was conducted to assess skill acquisition at the facility level during AMANAT Jyoti from baseline re-assessments to mid re-assessments.

We also fit mixed effect models with a random intercept for each facility to minimise loss of data due to missing timepoints. Data were available on each indicator at several, but not all, timepoints. These observations were clustered within facilities. As the indicators were dichotomous, we used a logistic regression model to estimate the odds ratios (OR). Simulations were performed once monthly during the initial AMANAT training, hence the ORs are reported as an increase in the odds of the individual skill for every month of simulation training. We used the "*melogit*" program in Stata to fit the models with a random intercept term for facility. Statistical analyses were conducted using SPSS version 23 [30] and Stata version 16 [31].

RESULTS

NR Skill Retention

PRONTO NR simulations were conducted at all three initial timepoints including AMANAT mid-assessments, AMANAT post-assessments, and AMANAT Jyoti baseline re-assessments at 64 PHCs across Bihar. This included 24 PHCs initially trained in AMANAT phase two, 14 in AMANAT phase three, and 26 in AMANAT phase four.

A significant increase in the percentage of facilities in which two key NR skills-- PPV with chest rise and heart rate assessment-- were performed correctly was observed over the initial PRONTO training period (AMANAT mid to post) followed by a decline over the training gap (AMANAT post to AMANAT Jyoti baseline; Table 1, Figure 2, $p<0.01$). For PPV with chest rise, a 31.1 percentage-point increase from mid- to post-initial training was observed ($p=0.03$) followed by a 26.2 percentage-point decrease over the training gap ($p=0.01$). For assessment of heart rate, an initial 13.1 percentage-point increase from mid- to post-training ($p=0.03$) was followed by a 20.9 percentage-point decrease over the training gap ($p<0.01$).

In all other skills assessed including stimulation, suction, neck extension, and rate of PPV delivery, there was no statistically significant changes over time. Finally, there was no statistically significant change from mid-assessments to baseline re-assessments for all skills including PPV with chest rise and heart rate assessment. Multilevel modeling showed consistent results where models converged (Supplementary Table).

NR Skill Re-Acquisition with Peer Simulation Facilitators

Of the 64 facilities that completed PRONTO assessments at all three initial timepoints, 40 had a fourth NR assessment simulation at the midpoint of AMANAT Jyoti. Additionally, five facilities that received PRONTO for the first time during AMANAT Jyoti, participated in both baseline re-assessment and mid re-assessment. Amongst these 45 facilities, a significant increase was observed in the percentage of facilities in which heart rate was assessed from baseline (79.5%) to mid (95.6%) re-assessment (Table 2, Figure 3, $p = 0.04$). There were no significant changes in the remaining skills assessed including stimulation, suction, neck extension, PPV with chest rise, and rate of PPV delivery (Table 2, Figure 3). Multilevel modeling showed consistent results (Supplementary Table).

Confidential: For Review Only

DISCUSSION

To be effective, NR requires not only knowledge of the resuscitation algorithm, but also technical competence in performing PPV and awareness of the need for continual re-evaluation of a neonate's clinical status, including assessment of heart rate. With PRONTO training, as part of the larger AMANAT nurse mentoring interventions in Bihar, an increase was observed in the proportion of PHCs in which PPV was delivered with effective chest rise and heart rate was assessed from mid to post training, consistent with a previous assessment conducted in 2017 [18]. However, this initial facility level improvement was followed by a decline in these same skills over a prolonged training gap. Nevertheless, under the new mentorship model of peer simulation facilitation employed in repeat PRONTO training as part of AMANAT Jyoti, the proportion of PHCs in which heart rate was assessed significantly improved again by mid re-assessments with only one NR simulation session.

To maintain technical NR skills, such as PPV, frequent repetition is key. Similar to this study, other studies have shown a decline in NR skills at one to six months post training without such repetition [9-14]. Programs that have achieved skill retention have in common the opportunity for frequent and ongoing NR practice through the time of skill re-assessment [14-16]. This study emphasises this need for frequent NR skill practice amongst frontline providers, who may not have these opportunities in their daily clinical work, despite the eight-month duration of initial PRONTO training within the AMANAT mentoring program. This need for repetition in NR training was underscored by AMANAT mentors in previous qualitative interviews due to the fact that evidence-based NR practices represent a significant departure from traditional clinical practices in Bihar [19, 20]. PPV and heart rate assessment are perhaps the most unfamiliar and technical skills of basic NR, thus it is not surprising that these skills were the ones to degrade over the training gap [32]. In a previous study, AMANAT mentors also identified supply issues, including availability of appropriately sized masks for ventilation bags and watches for keeping time, as additional barriers to PPV and heart rate assessment [18]. Although all necessary supplies were provided during simulation, lack of supply availability during the training gap may have contributed to the observed facility level skill decline by limiting practice opportunities.

Among the other skills assessed, no significant changes were observed at the facility level across all timepoints. It is possible that this was due to inadequate sample size, nevertheless, this pattern is overall consistent with a prior program assessments and hypotheses regarding this lack of change are discussed in detail elsewhere [18]. It is important to note that baseline assessments were not conducted prior to initial PRONTO training in AMANAT phases two through four. This was a deliberate choice to avoid bias from simulation artefact given mentees' lack of familiarity with simulation-based learning. However, based on previous qualitative analyses of mentors' evaluations of mentees' NR skills prior to training [18], it is likely that the AMANAT mid assessments represent a significant improvement compared to true baseline. Therefore, the lack of change observed in many NR skills in this study presumably reflects maintenance of acquired skills rather than lack of skill acquisition. Nevertheless, there is undoubtedly room for continued improvement.

This study had several limitations. As noted above, the small sample size may have precluded identification of significant changes in the performance of all NR skills. Additionally, this

Confidential: For Review Only

analysis assessed skill at a facility level. Due to high staff turnover at PHCs, not all ANM and GNM mentees participating in training under AMANAT Jyoti had previously received PRONTO training in AMANAT. As previously stated, the exact percentage of AMANAT Jyoti mentees who had received prior training was unknown. Assessing skill retention at the facility-level with this limitation risks over-estimating skill decline if nurses not previously trained enter PHCs in which training has already occurred. Another limitation was the difference in the beginning of the simulated NR scenario in AMANAT phases two and three, where the scenario started after birth, versus in AMANAT phase four and AMANAT Jyoti, where the scenario started with a normal delivery. To reduce the risk of bias, only skills required in both clinical scenarios were assessed and the time in which those skills were completed was not assessed despite the importance of urgency in NR. Finally, PRONTO simulation training was implemented as part of the AMANAT mentoring program as previously described. While we are unable to distinguish the individual impact of simulation and mentoring on performance in assessment simulations, this is not dissimilar to other simulation training programs which involve more components than simulation alone. This assessment is meant to be an assessment of the PRONTO program within the context of AMANAT mentoring.

The optimal frequency of basic NR simulation training for frontline providers in LMIC settings remains unknown [33, 34], with no current consensus except that it should occur more frequently than annually [33]. It is estimated that 5-10% of neonates will require basic resuscitation to transition after birth and, of that group, 3-6% will require bag-mask ventilation [35]. For frontline providers, this translates into very few opportunities to practice technical NR skills. Therefore, annual training is likely insufficient. Two recent studies on cardiopulmonary resuscitation (CPR), a similarly rare event requiring competency in technical skills, suggested repeat training at one-month intervals was optimal [36, 37]. In one study, providers were randomised to receive refresher training at one, three, six, or twelve-month intervals, and significantly improved performance was observed in the group that received monthly training compared to all other groups. Moreover, there was no difference in skill retention amongst groups who received refresher training at three, six, or 12-month intervals [37]. A monthly training interval is additionally supported by the fact that one month is the earliest published evidence of NR skill decline post simulation training in the LMIC literature [9]. Further, initial PRONTO training within AMANAT mentoring in Bihar occurred at monthly intervals over an eight-month training period and, at this interval, was demonstrated to improve NR skills in simulated and live deliveries [18].

Monthly training in an LMIC setting presents the unique challenge of sustainability. One possible solution is peer simulation facilitation. In this study, peer simulation facilitation was introduced in AMANAT Jyoti and was not only feasible but enabled re-acquisition of heart rate assessment skills by the midpoint of training, which occurred after only one NR simulation. Similarly, in Uganda peer-facilitated NR practice after an initial HBB training was demonstrated to promote maintenance of NR skills at six months post-training when peer facilitators received phone support from HBB trainers [14]. Finally, a randomised controlled trial in Syria demonstrated non-inferiority of peer-facilitated Basic Life Support training, which includes CPR, as compared to professionally-facilitated training [38]. Although more research is required into how to optimally train frontline healthcare providers to become experts in both clinical NR skills

Confidential: For Review Only

and simulation facilitation, limited existing evidence suggests peer instruction may offer both a successful and sustainable model of clinical instruction.

CONCLUSION

PRONTO simulation training implemented in Bihar as part of CARE India's AMANAT mentoring program in partnership with the local government, had a significant, positive impact on the delivery of effective PPV and neonatal heart rate assessment at the facility level. However, after a prolonged training gap, improvements in these skills were lost despite the relatively long duration of initial simulation training. Similar NR skill decline has been observed as early as one-month post-training in other LMIC settings, concordant with the limited opportunities frontline providers have to practice the technical skills required in NR. More frequent training is necessary, especially in settings where evidence-based NR skills may represent a large shift in clinical practice. In such contexts, a key challenge is the scale and sustainability of any NR training program, especially if monthly training is indicated. Employing peer simulation facilitators may be one way to address these issues.

List of abbreviations

NR: neonatal resuscitation
LMIC: low- and middle-income country
HBB: Helping Babies Breathe
PPV: positive pressure ventilation
PHC: primary health centre
ANM: auxiliary nurse midwife
GNM: general nurse midwife
UCSF: University of California San Francisco
OR: odds ratio
CPR: cardiopulmonary resuscitation

Funding

This study was supported by the Bill and Melinda Gates Foundation, grant number OPP1112431. The funding body had no role in study design, data collection, analysis, interpretation, manuscript writing, or the decision to submit the manuscript for publication.

Competing Interests

DW and SC are founding members of PRONTO International and sit on its board of directors. None of the other author have any competing interests to declare.

Authors' Contributions

BH led study design for this manuscript, analysed all simulation videos, performed the data analysis, and drafted and revised the manuscript. MM and HS contributed to study design, literature review, data interpretation and critical manuscript revision. RG, OL, and SC were also involved study design, data interpretation and provided critical manuscript revision. RG additionally provided input in data analysis and OL additionally provided on the ground oversight of training and local contextualization. AD, AG and TM were involved in study design, implementation, provided local contextualization, and provided critical input during

Confidential: For Review Only

manuscript revision. DW was the principal investigator and a major contributor to all aspects of this study and manuscript.

Availability of data and material
Data is not yet publicly available as the study is ongoing. Requests for data/materials may be made to the corresponding author.

Acknowledgements
The authors would like to thank CARE India as well as the nurse supervisors, nurse mentors and mentees working in the primary health centres throughout the state of Bihar for their hard work implementing and participating in both the AMANAT and AMANAT Jyoti nurse mentoring programs. Thank you also to the PRONTO Patna based staff members for facilitating simulation and team trainings and implementing the PRONTO curriculum in both programs as well as collecting, organizing, and managing hundreds of simulation videos. Lastly, we would like to thank Dr. Hemant Shah and the rest of the CARE India management for their unrelenting efforts to improve conditions for women and newborns in Bihar.

Key Words
Neonatal resuscitation
Simulation
Skill retention
Bihar
India

Confidential: For Review Only

REFERENCES

1. Collaborators GM. Global, regional, and national under-5 mortality, adult mortality, age-specific mortality, and life expectancy, 1970-2016: a systematic analysis for the Global Burden of Disease Study 2016. Lancet. 2017;390(10100):1084-150.
2. Liu L, Oza S, Hogan D, Chu Y, Perin J, Zhu J, et al. Global, regional, and national causes of under-5 mortality in 2000-15: an updated systematic analysis with implications for the Sustainable Development Goals. Lancet. 2016;388(10063):3027-35.
3. Reisman J, Arlington L, Jensen L, Louis H, Suarez-Rebling D, Nelson BD. Newborn Resuscitation Training in Resource-Limited Settings: A Systematic Literature Review. Pediatrics. 2016;138(2).
4. Kamath-Rayne BD, Berkelhamer SK, Kc A, Ersdal HL, Niermeyer S. Neonatal resuscitation in global health settings: an examination of the past to prepare for the future. Pediatr Res. 2017;82(2):194-200.
5. Dol J, Campbell-Yeo M, Murphy GT, Aston M, McMillan D, Richardson B. The impact of the Helping Babies Survive program on neonatal outcomes and health provider skills: a systematic review. JBI Database System Rev Implement Rep. 2018;16(3):701-37.
6. Enweronu-Laryea C, Dickson KE, Moxon SG, Simen-Kapeu A, Nyange C, Niermeyer S, et al. Basic newborn care and neonatal resuscitation: a multi-country analysis of health system bottlenecks and potential solutions. BMC Pregnancy Childbirth. 2015;15 Suppl 2:
7. WHO, UNICEF. 2014. *Every Newborn: an action plan to end preventable deaths*. Geneva: World Health Organization

8. Helping Babies Breathe. Available from: www.helpingbabiesbreathe.org [Accessed 15 March 2019].
9. Mildenberger C, Ellis C, Lee K. Neonatal resuscitation training for midwives in Uganda: Strengthening skill and knowledge retention. Midwifery. 2017;50:36-41.
10. Musafili A, Essén B, Baribwira C, Rukundo A, Persson L. Evaluating Helping Babies Breathe: training for healthcare workers at hospitals in Rwanda. Acta Paediatr. 2013;102(1):e34-8.
11. Eblovi D, Kelly P, Afua G, Agyapong S, Dante S, Pellerite M. Retention and use of newborn resuscitation skills following a series of helping babies breathe trainings for midwives in rural Ghana. Glob Health Action. 2017;10(1):1387985.
12. Carlo WA, Wright LL, Chomba E, McClure EM, Carlo ME, Bann CM, et al. Educational impact of the neonatal resuscitation program in low-risk delivery centres in a developing country. J Pediatr. 2009;154(4):504-8.e5.
13. Bang A, Patel A, Bellad R, Gisore P, Goudar SS, Esamai F, et al. Helping Babies Breathe (HBB) training: What happens to knowledge and skills over time? BMC Pregnancy Childbirth. 2016;16(1):364.
14. Evans CL, Bazant E, Atukunda I, Williams E, Niermeyer S, Hiner C, et al. Peer-assisted learning after onsite, low-dose, high-frequency training and practice on simulators to prevent and treat postpartum haemorrhage and neonatal asphyxia: A pragmatic trial in 12 districts in Uganda. PLoS One. 2018;13(12):e0207909.
15. Bookman L, Engmann C, Srofenyoh E, Enweronu-Laryea C, Owen M, Randolph G, et al. Educational impact of a hospital-based neonatal resuscitation program in Ghana. Resuscitation. 2010;81(9):1180-2.
16. Arabi AM, Ibrahim SA, Ahmed SE, MacGinnea F, Hawkes G, Dempsey E, et al. Skills retention in Sudanese village midwives 1 year following Helping Babies Breathe training. Arch Dis Child. 2016;101(5):439-42.
17. PRONTO International. Available from: www.prontointernational.org [Accessed 15 March 2019].
18. Vail B, Morgan MC, Spindler H, Christmas A, Cohen SR, Walker DM. The power of practice: simulation training improving the quality of neonatal resuscitation skills in Bihar, India. BMC Pediatr. 2018;18(1):291.
19. Vail B, Morgan MC, Dyer J, Christmas A, Cohen SR, Joshi M, et al. Logistical, cultural, and structural barriers to immediate neonatal care and neonatal resuscitation in Bihar, India. BMC Pregnancy Childbirth. 2018;18(1):385.
20. Morgan MC, Dyer J, Abril A, Christmas A, Mahapatra T, Das A, et al. Barriers and facilitators to the provision of optimal obstetric and neonatal emergency care and to the implementation of simulation-enhanced mentorship in primary care facilities in Bihar, India: a qualitative study. BMC Pregnancy Childbirth. 2018;18(1):420.
21. Registrar General of India. Population Enumeration Data. Available from: www.censusindia.gov.in/2011census/population_enumeration.html [Accessed 15 March 2019].
22. World Bank Group. Bihar: Poverty, Growth & Inequality. 2016 Available from: http://documents.worldbank.org/curated/en/781181467989480762/pdf/105842-BRI-P157572-PUBLIC-Bihar-Proverty.pdf [Accessed 15 March 2019].
23. Government of India Ministry of Health and Family Welfare. National Family Health Survey – 4. Available from: http://rchiips.org/nfhs/ [Accessed 15 March 2019].

Confidential: For Review Only

24. Health Management Information System portal: RCH reports. https://www.nrhm-mis.nic.in/hmisreports/frmstandard_reports.aspx [Accessed 29 January 2020].
25. Indian Nursing Council. Types of Nursing Programs. Available from: www.indiannursingcouncil.org/nursing-programs.asp?show=prog-type [Accessed 15 March 2019].
26. CARE India Monitoring and Evaluation Data. 2019. Unpublished.
27. CARE India. Available from: www.careindia.org [Accessed 15 March 2019].
28. Vail B, Spindler H, Morgan MC, Cohen SR, Christmas A, Sah P, et al. Care of the mother-infant dyad: a novel approach to conducting and evaluating neonatal resuscitation simulation training in Bihar, India. BMC Pregnancy Childbirth. 2017;17(1):252.
29. Neonatal Resuscitation Program. www.aap.org/en-us/continuing-medical-education/life-support/NRP/Pages/NRP.aspx [Accessed 15 March 2019].
30. IBM Corp. Released 2013. IBM SPSS statistics for Macintosh, version 23.0. Armonk: IBM Corp.
31. StataCorp. Stata Statistical Software: Release 16. College Station, TX: StataCorp LP. 2016.
32. Sawyer T, White M, Zaveri P, Chang T, Ades A, French H, et al. Learn, see, practice, prove, do, maintain: an evidence-based pedagogical framework for procedural skill training in medicine. Acad Med. 2015;90(8):1025-33.
33. Perlman JM, Wyllie J, Kattwinkel J, Wyckoff MH, Aziz K, Guinsburg R, et al. Part 7: Neonatal Resuscitation: 2015 International Consensus on Cardiopulmonary Resuscitation and Emergency Cardiovascular Care Science With Treatment Recommendations (Reprint). Pediatrics. 2015;136 Suppl 2:S120-66.
34. Sawyer T, Ades A, Ernst K, Colby C. Simulation and the Neonatal Resuscitation Program 7th Edition Curriculum. NeoReviews. 2016;17:e447.
35. Lee AC, Cousens S, Wall SN, Niermeyer S, Darmstadt GL, Carlo WA, et al. Neonatal resuscitation and immediate newborn assessment and stimulation for the prevention of neonatal deaths: a systematic review, meta-analysis and Delphi estimation of mortality effect. BMC Public Health.
2011;11(Suppl 3):S12.
36. Lin Y, Cheng A, Grant VJ, Currie GR, Hecker KG. Improving CPR quality with distributed practice and real-time feedback in pediatric healthcare providers - A randomised controlled trial. Resuscitation. 2018;130:6-12.
37. Anderson R, Sebaldt A, Lin Y, Cheng A. Optimal training frequency for acquisition and retention of high-quality CPR skills: A randomised trial. Resuscitation. 2019;135:153-61.
38. Abbas F, Sawaf B, Hanafi I, Hajeer MY, Zakaria MI, Abbas W, et al. Peers versus professional training of basic life support in Syria: a randomised controlled trial. BMC Med Educ. 2018;18(1):142.

Confidential: For Review Only

TABLES and FIGURES

Table 1. Percentage of facilities in which key skills were correctly performed in simulated neonatal resuscitations at mid-PRONTO training, post-training, and following a 15 to 29-month training gap (N = 192 assessment simulations at 64 facilities)

NR Skill	N[a]	AMANAT mid assessment[b]	AMANAT post assessment[c]	AMANAT Jyoti baseline re-assessment		Δ Mid -> Post[f]	Δ Post -> Baseline[f]	Δ Mid -> Baseline[f]
		% (n)[d]			*p*-value[e]	Δ %		
Stimulation	60	55.6 (35)	50.8 (31)	55.6 (35)	0.92	-4.8	4.8	0
Suction	58	63.9 (39)	67.8 (40)	67.2 (43)	0.82	3.9	-0.6	3.3
Neck extension	49	78.2 (43)	80.7 (46)	80.6 (50)	0.68	2.5	-0.1	2.4
PPV with chest rise	41	58.9 (33)	90.0 (45)	63.8 (37)	0.01	31.1*	-26.2*	4.9
Chest rise in ≤30 seconds	14	53.1 (17)	51.2 (22)	68.6 (24)	0.46	-1.9	17.4	15.5
Rate of PPV 40-60 bpm	53	36.7 (22)	41.4 (24)	38.7 (24)	0.88	4.7	-2.7	2.0
Heart rate assessed	54	85.2 (52)	98.3 (57)	77.4 (48)	<0.01	13.1*	-20.9*	-7.8

NR neonatal resuscitation; *PPV* positive pressure ventilation; *bpm* beats per minute

a. Total number of facilities in which NR skill could be assessed at all three timepoints mid through baseline, n varies due to video quality
b. Includes AMANAT phase 2, 3 and 4 mid-training assessments
c. Includes AMANAT phase 2, 3 and 4 post-training assessments
d. Percent (number) of facilities in which NR skill was performed correctly at a given timepoint
e. Cochran's Q test
f. * = significant change based on McNemar's test, p-values reported in text

Table 2. Percentage of facilities in which key skills were correctly performed in simulated neonatal resuscitations at baseline and mid-repeat PRONTO training with peer simulation facilitators (N = 90 assessment simulations at 45 facilities)

NR Skill	N[a]	AMANAT Jyoti baseline re-assessment	AMANAT Jyoti mid re-assessment	Δ Baseline -> Mid	
		% (n)[b]		Δ %	*p*-value[c]
Stimulation	44	52.3 (23)	62.2 (28)	9.9	0.45
Suction	45	71.1 (32)	66.7 (30)	-4.4	0.84
Neck extension	43	77.3 (34)	90.9 (40)	13.6	0.11
PPV with chest rise	43	65.1 (28)	77.8 (35)	12.7	0.15
Chest rise in ≤30s	21	65.4 (17)	46.9 (15)	-18.5	0.11
Rate of PPV 40-60 bpm	43	41.9 (18)	51.1 (23)	9.2	0.38
Heart rate assessed	44	79.5 (35)	95.6 (43)	16.1	0.04

NR neonatal resuscitation; *PPV* positive pressure ventilation; *bpm* beats per minute

a. Total number of facilities in which NR skill could be assessed at both timepoints, n varies due to video quality

Confidential: For Review Only

b. Percent (number) of facilities in which NR skill was performed correctly at a given timepoint
c. McNemar's test

Figure 1. Timing of AMANAT and AMANAT Jyoti training phases

Confidential: For Review Only

Figure 1. PRONTO training timeline and structure within AMANAT and AMANAT Jyoti

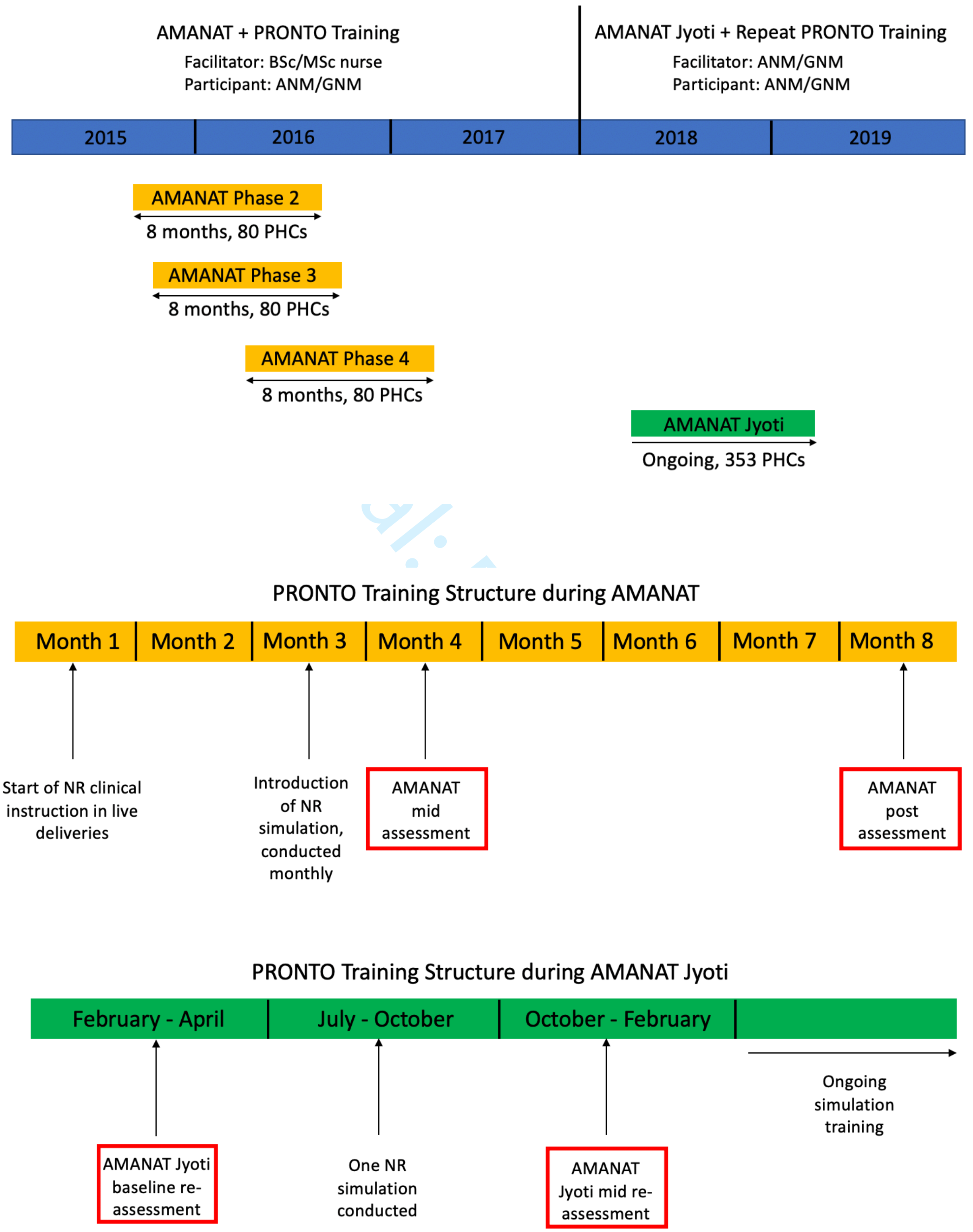

Supplemental Table. Mixed effects model of facility based neonatal resuscitation skill change with PRONTO training during AMANAT and AMANAT Jyoti

NR Skill	AMANAT mid[a] to AMANAT post[b]	*p*-value	AMANAT post[b] to AMANAT Jyoti baseline	*p*-value	AMANAT mid[a] to AMANAT Jyoti baseline	*p*-value	AMANAT Jyoti baseline to AMANAT Jyoti mid	*p*-value
	OR[c] (95% CI)		OR[c] (95% CI)		OR[c] (95% CI)		OR[c] (95% CI)	
Stimulation	0.84 (0.67, 1.06)	0.15	1.00 (0.97, 1.04)	0.91	1.00 (0.97, 1.04)	0.83	1.00 (0.91, 1.09)	0.96
Suction	1.02 (0.83, 1.25)	0.87	0.98 (0.94, 1.02)	0.38	0.99 (0.96, 1.03)	0.75	0.96 (0.90, 1.03)	0.22
Neck extension	1.04 (0.81, 1.33)	0.76	1.00 (0.96, 1.04)	0.98	1.00 (0.97, 1.04)	0.98	1.03 (0.92, 1.15)	0.64
PPV with chest rise	1.50 (1.15, 1.96)	<0.01	0.91 (0.86, 0.97)	<0.01	0.98 (0.94, 1.01)	0.19	1.01 (0.90, 1.13)	0.83
Chest rise in ≤30 seconds	1.01 (0.79, 1.28)	0.96	1.05 (0.98, 1.12)	0.15	1.04 (0.99, 1.09)	0.11	0.90 (0.81, 1.00)	0.05
Rate of PPV 40-60 bpm	1.13 (0.91, 1.42)	0.27	0.98 (0.95, 1.02)	0.30	0.99 (0.96, 1.02)	0.61	1.00 (0.93, 1.08)	0.90
Heart rate assessed	*	*	*	*	0.95 (0.91, 1.00)	0.04	1.22 (1.00, 1.48)	0.05

NR neonatal resuscitation; *OR* odds ratio; *CI* confidence interval; *PPV* positive pressure ventilation; *bpm* beats per minute

a. Includes AMANAT phase 2, 3 and 4 mid-training assessments
b. Includes AMANAT phase 2, 3 and 4 post-training assessments
c. Change in odds of the individual skill for every month of simulation training

* Models did not converge

Confidential: For Review Only

BMJ Paediatrics Open

A cohort study of neonatal resuscitation skill retention in frontline health care facilities in Bihar, India after PRONTO simulation training

Journal:	*BMJ Paediatrics Open*
Manuscript ID	bmjpo-2019-000628.R2
Article Type:	Original research
Date Submitted by the Author:	11-Mar-2020

Complete List of Authors:	Higgins, Brennan; University of California San Francisco, Pediatrics<br>Medvedev, Melissa; University of California San Francisco, Pediatrics<br>Spindler, Hilary; University of California San Francisco, Institute for Global Health Sciences<br>Ghosh, Rakesh; University of California San Francisco, Institute for Global Health Sciences<br>Longkumer, Ojungsangla; PRONTO International<br>Cohen, Susanna; University of Utah, College of Nursing<br>Das, Aritra; Care India<br>Gore, Aboli; Care India<br>Mahapatra, Tanmay; Care India<br>Walker, Dilys; University of California San Francisco, School of Medicine and Department of Obstetrics-Gynecology and Reproductive Sciences

Keywords:	Neonatology, Resuscitation

SCHOLARONE™
Manuscripts

I, the Submitting Author has the right to grant and does grant on behalf of all authors of the Work (as defined in the below author licence), an exclusive licence and/or a non-exclusive licence for contributions from authors who are: i) UK Crown employees; ii) where BMJ has agreed a CC-BY licence shall apply, and/or iii) in accordance with the terms applicable for US Federal Government officers or employees acting as part of their official duties; on a worldwide, perpetual, irrevocable, royalty-free basis to BMJ Publishing Group Ltd ("BMJ") its licensees and where the relevant Journal is co-owned by BMJ to the co-owners of the Journal, to publish the Work in this journal and any other BMJ products and to exploit all rights, as set out in our licence.

The Submitting Author accepts and understands that any supply made under these terms is made by BMJ to the Submitting Author unless you are acting as an employee on behalf of your employer or a postgraduate student of an affiliated institution which is paying any applicable article publishing charge ("APC") for Open Access articles. Where the Submitting Author wishes to make the Work available on an Open Access basis (and intends to pay the relevant APC), the terms of reuse of such Open Access shall be governed by a Creative Commons licence – details of these licences and which Creative Commons licence will apply to this Work are set out in our licence referred to above.

Other than as permitted in any relevant BMJ Author's Self Archiving Policies, I confirm this Work has not been accepted for publication elsewhere, is not being considered for publication elsewhere and does not duplicate material already published. I confirm all authors consent to publication of this Work and authorise the granting of this licence.

Confidential: For Review Only

A cohort study of neonatal resuscitation skill retention in frontline health care facilities in Bihar, India after PRONTO simulation training

Brennan V Higgins[1], Melissa M Medvedev[1,2], Hilary Spindler[3], Rakesh Ghosh[3], Ojungsangla Longkumer[4], Susanna R Cohen[5], Aritra Das[6], Aboli Gore[6], Tanmay Mahapatra[6], Dilys M Walker[7]

1. Department of Pediatrics, University of California San Francisco, 550 16th Street, 4th Floor, Box 0110, San Francisco, CA, 94158, USA
2. Maternal, Adolescent, Reproductive, and Child Health Centre, London School of Hygiene and Tropical Medicine, Keppel Street, London, WC1E 7HT, UK
3. Institute for Global Health Sciences, University of California San Francisco, 550 16th Street, San Francisco, CA 94158, USA
4. PRONTO International, State RMNCH+A, Patna, 80002, India
5. College of Nursing, University of Utah, 10 South 2000 East, Salt Lake City, UT, 84112, USA
6. CARE India, 14 Patliputra Colony, Patna, 800013, Bihar, India
7. School of Medicine and Department of Obstetrics-Gynecology and Reproductive Sciences, University of California San Francisco, 1001 Potrero Ave, San Francisco, CA, 94110, USA

Corresponding author: Brennan V Higgins
Address: Department of Pediatrics, University of California San Francisco, 550 16th Street, 4th Floor, Box 0110, San Francisco, CA 94143, USA
E-mail: brennan.vail@ucsf.edu
Telephone: 415-476-5001

Confidential: For Review Only

ABSTRACT

Background

Use of simulation in neonatal resuscitation (NR) training programs has increased throughout low- and middle-income countries. Many of such programs have demonstrated a positive impact on NR knowledge and skill acquisition along with reduction of early neonatal mortality and fresh stillbirth rates. However, NR skill retention after simulation programs remains a challenge.

Methods

This study assessed facility level NR skill retention after PRONTO International's simulation training in Bihar, India. Training was conducted within CARE India's statewide in-job, on-site AMANAT mentoring program as part of a larger quality improvement and health systems strengthening initiative. Public sector facilities were initially offered training, facilitated by trained nursing graduates, during eight-month phases between September 2015 and January 2017. Repeat training began in February 2018 and was facilitated by peers. NR skills in simulated resuscitations were assessed at the facility level at the mid- and endpoint of initial training and prior to and at the midpoint of repeat training.

Results

Facilities administering effective positive pressure ventilation (PPV) and assessing infant heart rate increased (31.1% and 13.1% respectively, both p=0.03) from mid- to post-initial training (n=64 PHCs and 192 simulations). This was followed by a 26.2% and 20.9% decline in these skills respectively over the training gap ($p \leq 0.01$). A significant increase (16.1%, $p = 0.04$) in heart rate assessment was observed by the midpoint of repeat training with peer facilitators (n=45 PHCs and 90 simulations). No significant change was observed in other skills assessed.

Conclusions

Despite initial improvement in select NR skills, deterioration was observed at a facility level post training. Given the technical nature of NR skills and the departure these skills represent from traditional practices in Bihar, refresher trainings at shorter intervals are likely necessary. Very limited evidence suggests peer simulation facilitators may enable such increased training frequency but further study is required.

What is known about the subject

- Neonatal resuscitation simulation training programs have spread throughout low- and middle-income settings; most with a model of brief periods (1-3 days) of instruction
- These training programs can improve provider knowledge and skill and thereby reduce early neonatal mortality and fresh stillbirths
- Skill retention after such training programs has been achieved only in programs with ongoing opportunities for training and practice

What this study adds

- This study evaluates skill retention after in-job, on site nurse mentoring with simulation training carried out over an eight-month period with the hypothesis that a longer duration of training may improve retention
- Despite a longer duration of initial training, skill deterioration was still observed post-training

Confidential: For Review Only

- Refresher trainings were possible with peer simulation facilitators, but demonstrated limited skill improvement among nurse mentees

INTRODUCTION

Neonatal mortality represents a disproportionate and unsolved public health problem globally [1], and intrapartum-related events remain a leading cause [2]. In response, neonatal resuscitation (NR) simulation training programs have proliferated in low- and middle-income countries (LMICs) [3, 4, 5]. Nevertheless, lack of skills among frontline providers remain a well-recognized barrier to immediate neonatal care [6] and a focus of the Every Newborn Action Plan, which aims to reduce neonatal mortality to 10 per 1000 live births in every country globally by 2035 [7]. Although NR training programs have already had a documented positive impact on knowledge and skill acquisition with subsequent reduction in early neonatal mortality and fresh stillbirths, skill retention after these programs remains a unique challenge and a key obstacle to the 2035 goal [3, 4, 5].

In LMICs, studies evaluating skill retention after NR simulation training programs have assessed retention from one to 12 months post-training. The majority of these studies assessed Helping Babies Breathe (HBB), a one to three-day simulation-based training program developed specifically for LMIC settings [8]. Other simulation training programs assessed were of comparable duration and content to HBB. Most of these studies reported some decline in NR skills after training completion, including at one month [9], three months [10], four months [11], and six months [12, 13, 14] post-training.

Notably, however, three studies did not observe uniform skill decay. One study observed maintenance of skills at nine to 12 months post-training, which the authors attributed to the unique study setting—a small group of providers (n=14) in a high-risk referral hospital in Accra, Ghana where providers perform NR daily [15]. A second study found a falloff in skills in two of three training groups at three to six months but maintenance of skills in the third group at this same timepoint [14]. In this latter group, simulators were left at the study facilities for ongoing practice, which was facilitated by clinical mentors who received phone support from district trainers. A final study saw improvements at three and 12-months post-training in all skills except positive pressure ventilation (PPV) [16]. In this study, facilities received the initial HBB training (two days) in addition to bi-weekly visits from trainers to encourage practice over the post-training period (12 months). Common to all three studies was the opportunity for frequent and ongoing practice. However, PPV was highlighted as an exception, with an observed decline in competence despite ongoing practice [16].

PRONTO International [17] developed a unique in-situ, intrapartum simulation training curriculum for LMIC settings, which was implemented phase-wise in Bihar, India over a long initial training period of approximately eight months per phase. The training was embedded in a state-wide mentoring program called "Apatkaleen Matritva evam Navjat Tatparta" (AMANAT) and implemented by CARE India in partnership with Government of Bihar. Assessment of PRONTO training implemented with AMANAT mentoring has demonstrated improved NR skills post-training in both simulated and live deliveries [18], despite the presence of many structural, logistical, and cultural barriers to neonatal care [19, 20]. However, NR skill retention

Confidential: For Review Only

following PRONTO training, or any simulation training of comparable duration in a LMIC setting, has not been assessed. The aim of this study was to assess retention of NR skills after PRONTO simulation training within the AMANAT mentoring program in Bihar for programmatic evaluation and possible application in similar LMIC settings. A secondary objective of this study was to assess skill re-acquisition with peer-facilitated simulations to better understand the feasibility of this approach to simulation training.

METHODS

Study setting

Training was conducted in Bihar, the third most populous state in the country of India [21]. According to the World Bank, more than one-third of Bihar's population lives below the international poverty line (<$1.90 per day) [22]. The documented neonatal mortality rate is 37 per 1000 live births [23], with significant under-reporting likely. This study assessed PRONTO training conducted alongside AMANAT mentoring in government basic emergency obstetric and neonatal care (BEmONC) facilities which were largely primary health centres (PHCs).

PHCs serve as entry-level facilities for preventative and basic medical care. Approximately 1.3 million deliveries are conducted annually in PHCs [24]. The delivery load per facility varies by location. Approximately 10% of mothers are referred from PHCs to higher-level facilities for delivery [25]. Deliveries in PHCs are attended by Auxiliary Nurse Midwives (ANM) or General Nurse Midwives (GNM), frontline providers with two or three and a half years of training after general education, respectively [26]. ANM/GNMs perform a variety of tasks at PHCs in addition to labour and delivery care. These often include other clinical obligations, including emergency care and outpatient care, as well as other tasks such as taking maintaining inventory supply and completing maternity registers. The average out-of-pocket expenditure of mothers in 2019 for a PHC delivery was 1788 Indian rupees, equivalent to 25 United States dollars. The majority of expenses resulted from diagnostic tests [25].

AMANAT and PRONTO interventions

AMANAT was a state-wide in-job, on-site mentoring program for obstetric and newborn care providers in public sector facilities. The program was implemented by CARE India [27] in collaboration with the government of Bihar as part of a health-system strengthening and quality of care improvement effort. PRONTO International [17] provided simulation training on obstetric and neonatal emergencies within the broader AMANAT initiative using the AMANAT mentor/mentee structure for clinical instruction. PRONTO training consists of in-situ, intrapartum simulations in addition to teamwork and communication exercises, skill stations, and case-based learning. The intrapartum nature of PRONTO training emphasises care of the mother-infant dyad, including complications that may co-exist such as NR and postpartum haemorrhage [28].

This study assessed PRONTO training conducted within the AMANAT initiative at PHCs between September 2015 and February 2019. The timeline of the intervention is depicted in Figure 1. From September 2015 to January 2017, mentoring and training were conducted in three equivalent eight-month phases (AMANAT phases two through four). AMANAT phase one was excluded from this analysis due to curricular and simulation assessment differences during that initial phase. Each phase reached 80 non-overlapping PHCs for a total of 240 PHCs. This was

Confidential: For Review Only

followed by a training gap prior to initiation of repeat PRONTO training as part of a second chapter of the AMANAT initiative called AMANAT Jyoti, which is a peer guided mentoring program aimed at sustainability. AMANAT Jyoti is currently ongoing. This second round of PRONTO training within AMANAT Jyoti reached 353 PHCs state-wide. The majority of these PHCs had received prior training/mentoring during AMANAT and thus AMANAT Jyoti marked the initiation of repeat PRONTO training. However, some of the PHCs where AMANAT Jyoti with refresher PRONTO training was rolled out were not included in the earlier PRONTO training and AMANAT mentoring program. Furthermore, even if the PHC had received prior simulation training and mentoring, due to staff turnover not all ANMs/GNMs in a given PHC at the time of AMANAT Jyoti had participated in the prior training and mentoring. The exact percentage of ANMs/GNMs who had received prior training is unknown.

During the AMANAT phase of the program, PRONTO NR simulations along with AMANAT mentoring were facilitated by nurses who were college educated (BSc and MSc) and recruited from across India. Mentees who received the simulation curriculum and mentoring were ANMs and GNMs posted in labor rooms in PHCs. ANM/GNMs were not paid to participate in PRONTO training or AMANAT mentoring and this was their first experience with simulation-based NR training. Some ANM/GNM mentees had participated in other governmental classroom-based trainings on topics including basic newborn care and NR. To ensure that AMANAT Jyoti had a sustainability model built into it, mentors, and thus simulation facilitators, transitioned to a cadre of trained ANMs and GNMs working in the Bihar PHC system. Mentees were peer ANM/GNMs.

Simulations and assessments

In AMANAT, nurse mentors worked in pairs under the supervision of a master nurse mentor to facilitate PRONTO's NR simulations at four PHCs, spending one week per month mentoring at each PHC. As depicted in Figure 1, NR simulations were conducted beginning in month three of mentoring and continued monthly through the end of the mentoring phase (month 8). NR clinical instruction during live deliveries began at the outset of the phase.

A single NR assessment simulation was conducted to assess skills at a facility level at month four ("AMANAT mid-assessment") and month eight ("AMANAT post-assessment") of training. Individual participants in the assessment simulation were chosen at random. Pre-training assessments were not conducted to give mentees time to adjust to simulation-based learning and thereby minimise the potential impact of simulation artefact on data. In AMANAT phases two and three, the NR assessment scenario began immediately after birth, whereas in phase four, the scenario also included a normal spontaneous vaginal delivery.

Prior to AMANAT Jyoti, a single NR simulation ("AMANAT Jyoti baseline re-assessment") was conducted in a random sample of PHCs (n=105) to assess skill retention at the facility level from AMANAT post-assessments. Following baseline re-assessments, peer NR simulation training along with mentoring was then introduced between July and October 2018. One simulation was conducted per PHC during this interval. Subsequently, AMANAT Jyoti mid re-assessments were conducted between October 2018 and February 2019 to assess NR skill acquisition, again at a facility level, with the new peer mentorship model. Both AMANAT Jyoti

Confidential: For Review Only

baseline re-assessment and mid re-assessment simulations began with a normal spontaneous vaginal delivery and subsequently required NR.

All assessment simulations were video-recorded for immediate debriefing and program assessment purposes.

Ethics approval

Ethical approval for this study was granted by the Committee on Human Research at University of California San Francisco (UCSF; 14-15446), the Institutional Committee for Ethics and Review of Research of the Indian Institute of Health Management Research in Jaipur, India for the training/ mentoring conducted during 2014-2015, and the Ashirwad Ethics Committee at the Ashirwad Hospital and Research Center in Ulhasnagar, India for the training/ mentoring conducted during and after 2016. All mentees provided consent for the use of video data in aggregated analyses.

Patient and public involvement

The data for this manuscript was simulation data and participants were frontline healthcare providers. Patients did not participate in this research. Results will be disseminated to local facilities through data dissemination efforts conducted collaboratively by PRONTO International, CARE India, and the Government of Bihar officials to assist with quality of care improvement efforts.

Analysis

NR assessment simulation videos from all phases were analysed by a Neonatal Resuscitation Program [29] certified physician at UCSF. Due to the minor differences in the simulated clinical scenario (with or without a preceding delivery), data could not be coded in a single blinded group. Assessment data was matched by facility across time to assess NR skill acquisition and retention at the facility level. The four assessment timepoints per PHC were AMANAT mid assessments, AMANAT post assessments, AMANAT Jyoti baseline re-assessments, and AMANANAT Jyoti mid re-assessments. The training gap between AMANAT post assessments and AMANAT Jyoti baseline re-assessments allowed for assessment of NR skill retention at a facility level. The duration of this gap ranged from 15 to 29 months depending on the AMANAT phase in which the PHC originally participated (Figure 1).

A Cochran's Q test was conducted to assess if there was any difference across matched facilities in the percentage of facilities in which key NR skills were correctly performed at any time from AMANAT mid assessments to AMANAT Jyoti baseline re-assessments. Due to the matched nature of this test, only facilities where skills could be evaluated at all timepoints (mid assessment, post assessment, and baseline re-assessment) were included in the statistical analysis. The number of facilities included varied by skill as not all skills could be evaluated in every simulation video due to video quality issues. For skills that demonstrated a statistically significant change over time, McNemar's test was used to individually assess change from AMANAT mid to post assessments, AMANAT post assessments to AMANAT Jyoti baseline re-assessments, and AMANAT mid assessments to AMANAT Jyoti baseline re-assessments. Additionally, a McNemar's test was conducted to assess skill acquisition at the facility level during AMANAT Jyoti from baseline re-assessments to mid re-assessments.

Confidential: For Review Only

We also fit mixed effect models with a random intercept for each facility to minimise loss of data due to missing timepoints. Data were available on each indicator at several, but not all, timepoints. Individual observations were clustered within facilities, giving it a two-level data structure. As the indicators were dichotomous, we used a logistic regression model to estimate the odds ratios (OR). Simulations were performed once monthly during the initial AMANAT training, hence the associations are reported as an increase in the odds of the individual skills for every month of simulation training. We used the "*melogit*" program in Stata to fit the models with a random intercept term for facility. Statistical analyses were conducted using SPSS version 23 [30] and Stata version 16 [31].

RESULTS

NR Skill Retention

PRONTO NR simulations were conducted at all three initial timepoints including AMANAT mid-assessments, AMANAT post-assessments, and AMANAT Jyoti baseline re-assessments at 64 PHCs across Bihar. This included 24 PHCs initially trained in AMANAT phase two, 14 in AMANAT phase three, and 26 in AMANAT phase four.

A significant increase in the percentage of facilities in which two key NR skills-- PPV with chest rise and heart rate assessment-- were performed correctly was observed over the initial training period (AMANAT mid to post) followed by a decline over the training gap (AMANAT post to AMANAT Jyoti baseline; Table 1, Figure 2, p<0.01). For PPV with chest rise, a 31.1 percentage-point increase from mid- to post-initial training was observed (p=0.03) followed by a 26.2 percentage-point decrease over the training gap (p=0.01). For assessment of heart rate, an initial 13.1 percentage-point increase from mid- to post-training (p=0.03) was followed by a 20.9 percentage-point decrease over the training gap (p<0.01).

In all other skills assessed including stimulation, suction, neck extension, and rate of PPV delivery, there was no statistically significant changes over time. Finally, there was no statistically significant change from mid-assessments to baseline re-assessments for all skills including PPV with chest rise and heart rate assessment.

The findings above were confirmed by multilevel models, where convergence was achieved (Table 3). From AMANAT mid to post, there was an increased in odds of correctly performing PPV with chest rise per month of simulation training (OR 1.50, 95% CI 1.15-1.96, p < 0.01). However, from AMANAT post to AMANAT Jyoti baseline when there was a gap in training, a lower odds per month passed was observed (OR 0.91, 95% CI 0.86-0.97, p < 0.01). Results for heart rate assessment were similar for the time points AMANAT mid to AMANAT Jyoti baseline, where a small decrease in odds of performing this skill was observed (OR 0.95, 95% CI 0.91- 0.99, p=0.04).

NR Skill Re-Acquisition with Peer Simulation Facilitators

Of the 64 facilities that completed NR assessments at all three initial timepoints, 40 had a fourth NR assessment simulation at the midpoint of AMANAT Jyoti. Additionally, five facilities that received simulation training and mentoring for the first time during AMANAT Jyoti, participated

Confidential: For Review

in both baseline re-assessment and mid re-assessment. Amongst these 45 facilities, a significant increase was observed in the percentage of facilities in which heart rate was assessed from baseline (79.5%) to mid (95.6%) re-assessment (Table 2, $p = 0.04$). There were no statistically significant changes in the remaining skills assessed including stimulation, suction, neck extension, PPV with chest rise, and rate of PPV delivery (Table 2).

Multilevel modeling also demonstrated a higher odds of heart rate assessment from AMANAT Jyoti baseline to AMANAT Jyoti mid re-assessment (Table 3, OR 1.22, 95% CI 1.00-1.48, p=0.05). There was no statistically significant change in the odds of performing PPV with chest rise for these two timepoints. However, of those facilities where chest rise was achieved, there was a small decrease in odds of it being achieved within ≤ 30 seconds (Table 3, OR 0.90, 95% CI 0.81-0.99, p=0.05).

DISCUSSION

To be effective, NR requires not only knowledge of the resuscitation algorithm, but also technical competence in performing PPV and awareness of the need for continual re-evaluation of a neonate's clinical status, including assessment of heart rate. With PRONTO training, as part of the larger AMANAT nurse mentoring interventions in Bihar, an increase was observed in the proportion of PHCs in which PPV was delivered with effective chest rise and heart rate was assessed from mid to post training, consistent with a previous assessment conducted in 2017 [18]. However, this initial facility level improvement was followed by a decline in these same skills over a prolonged training gap. Nevertheless, under the new mentorship model of peer simulation facilitation employed in repeat PRONTO training as part of AMANAT Jyoti, the proportion of PHCs in which heart rate was assessed significantly improved again by mid re-assessments with only one NR simulation session.

To maintain technical NR skills, such as PPV, frequent repetition is key. Similar to this study, other studies have shown a decline in NR skills at one to six months post training without such repetition [9-14]. Programs that have achieved skill retention have in common the opportunity for frequent and ongoing NR practice through the time of skill re-assessment [14-16]. This study emphasises this need for frequent NR skill practice amongst frontline providers, who may not have these opportunities in their daily clinical work, despite the eight-month duration of initial PRONTO training within the AMANAT mentoring program. This need for repetition in NR training was underscored by AMANAT mentors in previous qualitative interviews due to the fact that evidence-based NR practices represent a significant departure from traditional clinical practices in Bihar [19, 20]. PPV and heart rate assessment are perhaps the most unfamiliar and technical skills of basic NR, thus it is not surprising that these skills were the ones to degrade over the training gap [32]. In a previous study, AMANAT mentors also identified supply issues, including availability of appropriately sized masks for ventilation bags and watches for keeping time, as additional barriers to PPV and heart rate assessment [18]. Although all necessary supplies were provided during simulation, lack of supply availability during the training gap may have contributed to the observed facility level skill decline by limiting practice opportunities.

Among the other skills assessed, no significant changes were observed at the facility level across all timepoints. It is possible that this was due to inadequate sample size, nevertheless, this pattern is overall consistent with a prior program assessments and hypotheses regarding this lack of

Confidential: For Review Only

change are discussed in detail elsewhere [18]. It is important to note that baseline assessments were not conducted prior to initial PRONTO training in AMANAT phases two through four. This was a deliberate choice to avoid bias from simulation artefact given mentees' lack of familiarity with simulation-based learning. However, based on previous qualitative analyses of mentors' evaluations of mentees' NR skills prior to training [18], it is likely that the AMANAT mid assessments represent a significant improvement compared to true baseline. Therefore, the lack of change observed in many NR skills in this study presumably reflects maintenance of acquired skills rather than lack of skill acquisition. Nevertheless, there is undoubtedly room for continued improvement.

This study had several limitations. As noted above, the small sample size may have precluded identification of significant changes in the performance of all NR skills. Additionally, this analysis assessed skill at a facility level. Due to high staff turnover at PHCs, not all ANM and GNM mentees participating in training under AMANAT Jyoti had previously received PRONTO training under the AMANAT mentoring program. As previously stated, the exact percentage of AMANAT Jyoti mentees who had received prior training was unknown. Assessing skill retention at the facility-level with this limitation risks over-estimating skill decline if nurses not previously trained enter PHCs in which training has already occurred. Another limitation was the difference in the beginning of the simulated NR scenario in AMANAT phases two and three, where the scenario started after birth, versus in AMANAT phase four and AMANAT Jyoti, where the scenario started with a normal delivery. To reduce the risk of bias, only skills required in both clinical scenarios were assessed and the time in which those skills were completed was not assessed despite the importance of urgency in NR. Finally, PRONTO simulation training was implemented as part of the AMANAT mentoring program as previously described. While we are unable to distinguish the individual impact of simulation and mentoring on performance in assessment simulations, this is not dissimilar to other simulation training programs which involve more components than simulation alone. This assessment is meant to be an assessment of the PRONTO program within the context of AMANAT mentoring.

The optimal frequency of basic NR simulation training for frontline providers in LMIC settings remains unknown [33, 34], with no current consensus except that it should occur more frequently than annually [33]. It is estimated that 5-10% of neonates will require basic resuscitation to transition after birth and, of that group, 3-6% will require bag-mask ventilation [35]. For frontline providers, this translates into very few opportunities to practice technical NR skills. Therefore, annual training is likely insufficient. Two recent studies on cardiopulmonary resuscitation (CPR), a similarly rare event requiring competency in technical skills, suggested repeat training at one-month intervals was optimal [36, 37]. In one study, providers were randomised to receive refresher training at one, three, six, or twelve-month intervals, and significantly improved performance was observed in the group that received monthly training compared to all other groups. Moreover, there was no difference in skill retention amongst groups who received refresher training at three, six, or 12-month intervals [37]. A monthly training interval is additionally supported by the fact that one month is the earliest published evidence of NR skill decline post simulation training in the LMIC literature [9]. Further, initial PRONTO training within AMANAT mentoring in Bihar occurred at monthly intervals over an eight-month training period and, at this interval, was demonstrated to improve NR skills in simulated and live deliveries [18].

Confidential: For Review Only

Monthly training using external trainers or mentors in an LMIC setting presents the unique challenge of sustainability. One possible solution is peer simulation facilitation. In this study, peer simulation facilitation was introduced in AMANAT Jyoti. Programmatically, it was feasible and early evidence suggests it enabled re-acquisition of heart rate assessment skills by the midpoint of training. Although improvement was limited to one NR skill, this occurred with only one peer-facilitated simulation. Peer simulation facilitation has been studied in other low-resource settings including Uganda where peer-facilitated NR practice after an initial HBB training was demonstrated to promote maintenance of NR skills at six months post-training when peer facilitators received phone support from HBB trainers [14]. Additionally, a randomised controlled trial in Syria demonstrated non-inferiority of peer-facilitated Basic Life Support training, which includes CPR, as compared to professionally-facilitated training [38]. Although more research is required into how to optimally train frontline healthcare providers to become experts in both clinical NR skills and simulation facilitation, limited existing evidence suggests peer instruction may offer both a successful and sustainable model of clinical instruction.

CONCLUSION

PRONTO simulation training implemented in Bihar as part of CARE India's AMANAT mentoring program in partnership with the local government, had a significant, positive impact on the delivery of effective PPV and neonatal heart rate assessment at the facility level. However, after a prolonged training gap, improvements in these skills were lost despite the relatively long duration of initial simulation training. Similar NR skill decline has been observed as early as one-month post-training in other LMIC settings, concordant with the limited opportunities frontline providers have to practice the technical skills required in NR. More frequent training is necessary, especially in settings where evidence-based NR skills may represent a large shift in clinical practice. In such contexts, a key challenge is the scale and sustainability of any NR training program, especially if monthly training is indicated. With further research, peer simulation facilitators may be one way to address these issues.

List of abbreviations

NR: neonatal resuscitation
LMIC: low- and middle-income country
HBB: Helping Babies Breathe
PPV: positive pressure ventilation
PHC: primary health centre
ANM: auxiliary nurse midwife
GNM: general nurse midwife
UCSF: University of California San Francisco
OR: odds ratio
CPR: cardiopulmonary resuscitation

Funding

This study was supported by the Bill and Melinda Gates Foundation, grant number OPP1112431. The funding body had no role in study design, data collection, analysis, interpretation, manuscript writing, or the decision to submit the manuscript for publication.

Confidential: For Review Only

Competing Interests
DW and SC are founding members of PRONTO International and sit on its board of directors. None of the other author have any competing interests to declare.

Authors' Contributions
BH led study design for this manuscript, analysed all simulation videos, performed the data analysis, and drafted and revised the manuscript. MM and HS contributed to study design, literature review, data interpretation and critical manuscript revision. RG, OL, and SC were also involved study design, data interpretation and provided critical manuscript revision. RG additionally provided input in data analysis and OL additionally provided on the ground oversight of training and local contextualization. AD, AG and TM were involved in study design, implementation, provided local contextualization, and provided critical input during manuscript revision. DW was the principal investigator and a major contributor to all aspects of this study and manuscript.

Availability of data and material
Data is not yet publicly available as the study is ongoing. Requests for data/materials may be made to the corresponding author.

Acknowledgements
The authors would like to thank CARE India as well as the nurse supervisors, nurse mentors and mentees working in the primary health centres throughout the state of Bihar for their hard work implementing and participating in both the AMANAT and AMANAT Jyoti nurse mentoring programs. Thank you also to the PRONTO Patna based staff members for facilitating simulation and team trainings and implementing the PRONTO curriculum in both programs as well as collecting, organizing, and managing hundreds of simulation videos. Lastly, we would like to thank Dr. Hemant Shah and the rest of the CARE India management for their unrelenting efforts to improve conditions for women and newborns in Bihar.

Key Words
Neonatal resuscitation
Simulation
Skill retention
Bihar
India

REFERENCES

1. Collaborators GM. Global, regional, and national under-5 mortality, adult mortality, age-specific mortality, and life expectancy, 1970-2016: a systematic analysis for the Global Burden of Disease Study 2016. Lancet. 2017;390(10100):1084-150.
2. Liu L, Oza S, Hogan D, Chu Y, Perin J, Zhu J, et al. Global, regional, and national causes of under-5 mortality in 2000-15: an updated systematic analysis with implications for the Sustainable Development Goals. Lancet. 2016;388(10063):3027-35.

Confidential: For Review Only

3. Reisman J, Arlington L, Jensen L, Louis H, Suarez-Rebling D, Nelson BD. Newborn Resuscitation Training in Resource-Limited Settings: A Systematic Literature Review. Pediatrics. 2016;138(2).
4. Kamath-Rayne BD, Berkelhamer SK, Kc A, Ersdal HL, Niermeyer S. Neonatal resuscitation in global health settings: an examination of the past to prepare for the future. Pediatr Res. 2017;82(2):194-200.
5. Dol J, Campbell-Yeo M, Murphy GT, Aston M, McMillan D, Richardson B. The impact of the Helping Babies Survive program on neonatal outcomes and health provider skills: a systematic review. JBI Database System Rev Implement Rep. 2018;16(3):701-37.
6. Enweronu-Laryea C, Dickson KE, Moxon SG, Simen-Kapeu A, Nyange C, Niermeyer S, et al. Basic newborn care and neonatal resuscitation: a multi-country analysis of health system bottlenecks and potential solutions. BMC Pregnancy Childbirth. 2015;15 Suppl 2:
7. WHO, UNICEF. 2014. *Every Newborn: an action plan to end preventable deaths*. Geneva: World Health Organization
8. Helping Babies Breathe. Available from: www.helpingbabiesbreathe.org [Accessed 15 March 2019].
9. Mildenberger C, Ellis C, Lee K. Neonatal resuscitation training for midwives in Uganda: Strengthening skill and knowledge retention. Midwifery. 2017;50:36-41.
10. Musafili A, Essén B, Baribwira C, Rukundo A, Persson L. Evaluating Helping Babies Breathe: training for healthcare workers at hospitals in Rwanda. Acta Paediatr. 2013;102(1):e34-8.
11. Eblovi D, Kelly P, Afua G, Agyapong S, Dante S, Pellerite M. Retention and use of newborn resuscitation skills following a series of helping babies breathe trainings for midwives in rural Ghana. Glob Health Action. 2017;10(1):1387985.
12. Carlo WA, Wright LL, Chomba E, McClure EM, Carlo ME, Bann CM, et al. Educational impact of the neonatal resuscitation program in low-risk delivery centres in a developing country. J Pediatr. 2009;154(4):504-8.e5.
13. Bang A, Patel A, Bellad R, Gisore P, Goudar SS, Esamai F, et al. Helping Babies Breathe (HBB) training: What happens to knowledge and skills over time? BMC Pregnancy Childbirth. 2016;16(1):364.
14. Evans CL, Bazant E, Atukunda I, Williams E, Niermeyer S, Hiner C, et al. Peer-assisted learning after onsite, low-dose, high-frequency training and practice on simulators to prevent and treat postpartum haemorrhage and neonatal asphyxia: A pragmatic trial in 12 districts in Uganda. PLoS One. 2018;13(12):e0207909.
15. Bookman L, Engmann C, Srofenyoh E, Enweronu-Laryea C, Owen M, Randolph G, et al. Educational impact of a hospital-based neonatal resuscitation program in Ghana. Resuscitation. 2010;81(9):1180-2.
16. Arabi AM, Ibrahim SA, Ahmed SE, MacGinnea F, Hawkes G, Dempsey E, et al. Skills retention in Sudanese village midwives 1 year following Helping Babies Breathe training. Arch Dis Child. 2016;101(5):439-42.
17. PRONTO International. Available from: www.prontointernational.org [Accessed 15 March 2019].
18. Vail B, Morgan MC, Spindler H, Christmas A, Cohen SR, Walker DM. The power of practice: simulation training improving the quality of neonatal resuscitation skills in Bihar, India. BMC Pediatr. 2018;18(1):291.

Confidential: For Review Only

19. Vail B, Morgan MC, Dyer J, Christmas A, Cohen SR, Joshi M, et al. Logistical, cultural, and structural barriers to immediate neonatal care and neonatal resuscitation in Bihar, India. BMC Pregnancy Childbirth. 2018;18(1):385.
20. Morgan MC, Dyer J, Abril A, Christmas A, Mahapatra T, Das A, et al. Barriers and facilitators to the provision of optimal obstetric and neonatal emergency care and to the implementation of simulation-enhanced mentorship in primary care facilities in Bihar, India: a qualitative study. BMC Pregnancy Childbirth. 2018;18(1):420.
21. Registrar General of India. Population Enumeration Data. Available from: www.censusindia.gov.in/2011census/population_enumeration.html [Accessed 15 March 2019].
22. World Bank Group. Bihar: Poverty, Growth & Inequality. 2016 Available from: http://documents.worldbank.org/curated/en/781181467989480762/pdf/105842-BRI-P157572-PUBLIC-Bihar-Proverty.pdf [Accessed 15 March 2019].
23. Government of India Ministry of Health and Family Welfare. National Family Health Survey – 4. Available from: http://rchiips.org/nfhs/ [Accessed 15 March 2019].
24. Health Management Information System portal: RCH reports. https://www.nrhm-mis.nic.in/hmisreports/frmstandard_reports.aspx [Accessed 29 January 2020].
25. Concurrent Measurement and Learning Unit of CARE India, Bihar Technical Support Program, 2019. Unpublished primary data.
26. Indian Nursing Council. Types of Nursing Programs. Available from: www.indiannursingcouncil.org/nursing-programs.asp?show=prog-type [Accessed 15 March 2019].
27. CARE India. Available from: www.careindia.org [Accessed 15 March 2019].
28. Vail B, Spindler H, Morgan MC, Cohen SR, Christmas A, Sah P, et al. Care of the mother-infant dyad: a novel approach to conducting and evaluating neonatal resuscitation simulation training in Bihar, India. BMC Pregnancy Childbirth. 2017;17(1):252.
29. Neonatal Resuscitation Program. www.aap.org/en-us/continuing-medical-education/life-support/NRP/Pages/NRP.aspx [Accessed 15 March 2019].
30. IBM Corp. Released 2013. IBM SPSS statistics for Macintosh, version 23.0. Armonk: IBM Corp.
31. StataCorp. Stata Statistical Software: Release 16. College Station, TX: StataCorp LP. 2016.
32. Sawyer T, White M, Zaveri P, Chang T, Ades A, French H, et al. Learn, see, practice, prove, do, maintain: an evidence-based pedagogical framework for procedural skill training in medicine. Acad Med. 2015;90(8):1025-33.
33. Perlman JM, Wyllie J, Kattwinkel J, Wyckoff MH, Aziz K, Guinsburg R, et al. Part 7: Neonatal Resuscitation: 2015 International Consensus on Cardiopulmonary Resuscitation and Emergency Cardiovascular Care Science With Treatment Recommendations (Reprint). Pediatrics. 2015;136 Suppl 2:S120-66.
34. Sawyer T, Ades A, Ernst K, Colby C. Simulation and the Neonatal Resuscitation Program 7th Edition Curriculum. NeoReviews. 2016;17:e447.
35. Lee AC, Cousens S, Wall SN, Niermeyer S, Darmstadt GL, Carlo WA, et al. Neonatal resuscitation and immediate newborn assessment and stimulation for the prevention of neonatal deaths: a systematic review, meta-analysis and Delphi estimation of mortality effect. BMC Public Health. 2011;11(Suppl 3):S12.

Confidential: For Review Only

36. Lin Y, Cheng A, Grant VJ, Currie GR, Hecker KG. Improving CPR quality with distributed practice and real-time feedback in pediatric healthcare providers - A randomised controlled trial. Resuscitation. 2018;130:6-12.
37. Anderson R, Sebaldt A, Lin Y, Cheng A. Optimal training frequency for acquisition and retention of high-quality CPR skills: A randomised trial. Resuscitation. 2019;135:153-61.
38. Abbas F, Sawaf B, Hanafi I, Hajeer MY, Zakaria MI, Abbas W, et al. Peers versus professional training of basic life support in Syria: a randomised controlled trial. BMC Med Educ. 2018;18(1):142.

TABLES and FIGURES

Table 1. Percentage of facilities in which key skills were correctly performed in simulated neonatal resuscitations at mid-PRONTO training, post-training, and following a 15 to 29-month training gap (N = 192 assessment simulations at 64 facilities)

NR Skill	N[a]	AMANAT mid assessment[b]	AMANAT post assessment[c]	AMANAT Jyoti baseline re-assessment		Δ Mid -> Post[f]	Δ Post -> Baseline[f]	Δ Mid -> Baseline[f]
		% (n)[d]			*p*-value[e]	Δ %		
Stimulation	60	55.6 (35)	50.8 (31)	55.6 (35)	0.92	-4.8	4.8	0
Suction	58	63.9 (39)	67.8 (40)	67.2 (43)	0.82	3.9	-0.6	3.3
Neck extension	49	78.2 (43)	80.7 (46)	80.6 (50)	0.68	2.5	-0.1	2.4
PPV with chest rise	41	58.9 (33)	90.0 (45)	63.8 (37)	0.01	31.1*	-26.2*	4.9
Chest rise in ≤30 seconds	14	53.1 (17)	51.2 (22)	68.6 (24)	0.46	-1.9	17.4	15.5
Rate of PPV 40-60 bpm	53	36.7 (22)	41.4 (24)	38.7 (24)	0.88	4.7	-2.7	2.0
Heart rate assessed	54	85.2 (52)	98.3 (57)	77.4 (48)	<0.01	13.1*	-20.9*	-7.8

NR neonatal resuscitation; *PPV* positive pressure ventilation; *bpm* beats per minute

a. Total number of facilities in which NR skill could be assessed at all three timepoints mid through baseline, n varies due to video quality
b. Includes AMANAT phase 2, 3 and 4 mid-training assessments
c. Includes AMANAT phase 2, 3 and 4 post-training assessments
d. Percent (number) of facilities in which NR skill was performed correctly at a given timepoint
e. Cochran's Q test
f. * = significant change based on McNemar's test, p-values reported in text

Confidential: For Review Only

Table 2. Percentage of facilities in which key skills were correctly performed in simulated neonatal resuscitations at baseline and mid-repeat PRONTO training with peer simulation facilitators (N = 90 assessment simulations at 45 facilities)

NR Skill	N[a]	AMANAT Jyoti baseline re-assessment	AMANAT Jyoti mid re-assessment	Δ Baseline -> Mid	
		% (n)[b]		Δ %	*p*-value[c]
Stimulation	44	52.3 (23)	62.2 (28)	9.9	0.45
Suction	45	71.1 (32)	66.7 (30)	-4.4	0.84
Neck extension	43	77.3 (34)	90.9 (40)	13.6	0.11
PPV with chest rise	43	65.1 (28)	77.8 (35)	12.7	0.15
Chest rise in ≤30s	21	65.4 (17)	46.9 (15)	-18.5	0.11
Rate of PPV 40-60 bpm	43	41.9 (18)	51.1 (23)	9.2	0.38
Heart rate assessed	44	79.5 (35)	95.6 (43)	16.1	0.04

NR neonatal resuscitation; *PPV* positive pressure ventilation; *bpm* beats per minute

a. Total number of facilities in which NR skill could be assessed at both timepoints, n varies due to video quality
b. Percent (number) of facilities in which NR skill was performed correctly at a given timepoint
c. McNemar's test

Table 3. Mixed effects model of facility based neonatal resuscitation skill change with PRONTO training during AMANAT and AMANAT Jyoti

NR Skill	AMANAT mid[a] to AMANAT post[b]	*p*-value	AMANAT post[b] to AMANAT Jyoti baseline	*p*-value	AMANAT mid[a] to AMANAT Jyoti baseline	*p*-value	AMANAT Jyoti baseline to AMANAT Jyoti mid	*p*-value
	OR[c] (95% CI)		OR[c] (95% CI)		OR[c] (95% CI)		OR[c] (95% CI)	
Stimulation	0.84 (0.67, 1.06)	0.15	1.00 (0.97, 1.04)	0.91	1.00 (0.97, 1.04)	0.83	1.00 (0.91, 1.09)	0.96
Suction	1.02 (0.83, 1.25)	0.87	0.98 (0.94, 1.02)	0.38	0.99 (0.96, 1.03)	0.75	0.96 (0.90, 1.03)	0.22
Neck extension	1.04 (0.81, 1.33)	0.76	1.00 (0.96, 1.04)	0.98	1.00 (0.97, 1.04)	0.98	1.03 (0.92, 1.15)	0.64
PPV with chest rise	1.50 (1.15, 1.96)	<0.01	0.91 (0.86, 0.97)	<0.01	0.98 (0.94, 1.01)	0.19	1.01 (0.90, 1.13)	0.83
Chest rise in ≤30 seconds	1.01 (0.79, 1.28)	0.96	1.05 (0.98, 1.12)	0.15	1.04 (0.99, 1.09)	0.11	0.90 (0.81, 1.00)	0.05
Rate of PPV 40-60 bpm	1.13 (0.91, 1.42)	0.27	0.98 (0.95, 1.02)	0.30	0.99 (0.96, 1.02)	0.61	1.00 (0.93, 1.08)	0.90
Heart rate assessed	*	*	*	*	0.95 (0.91, 1.00)	0.04	1.22 (1.00, 1.48)	0.05

NR neonatal resuscitation; *OR* odds ratio; *CI* confidence interval; *PPV* positive pressure ventilation; *bpm* beats per minute

a. Includes AMANAT phase 2, 3 and 4 mid-training assessments
b. Includes AMANAT phase 2, 3 and 4 post-training assessments
c. Change in odds of the individual skill per month

* Models did not converge

Confidential: For Review Only

Figure 1. Timing of AMANAT and AMANAT Jyoti training phases

Confidential: For Review Only

Figure 1. PRONTO training timeline and structure within AMANAT and AMANAT Jyoti

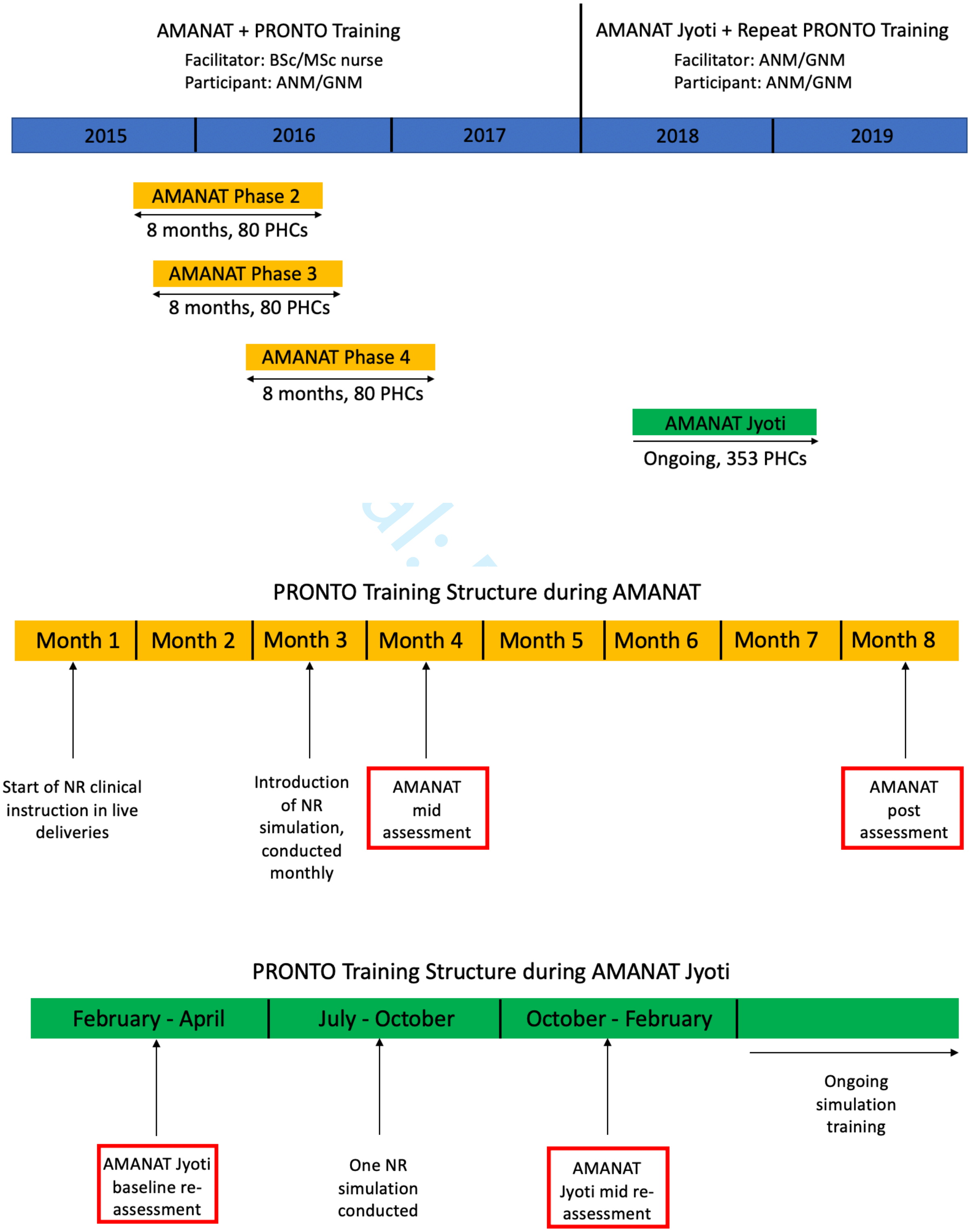

Supplement: Author's manuscript [file bmjpo-2019-000628.draft_revisions.pdf]
